# “Lock and Key” and “Induced-Fit” Host–Guest Models in Two Digold(I)-Based Metallotweezers

**DOI:** 10.1021/acs.inorgchem.2c00677

**Published:** 2022-04-01

**Authors:** Susana Ibáñez, Eduardo Peris

**Affiliations:** † Institute of Advanced Materials, Centro de Innovación en Química Avanzada, Universitat Jaume I, Avenida Vicente Sos Baynat s/n, Castellón E-12071, Spain

## Abstract

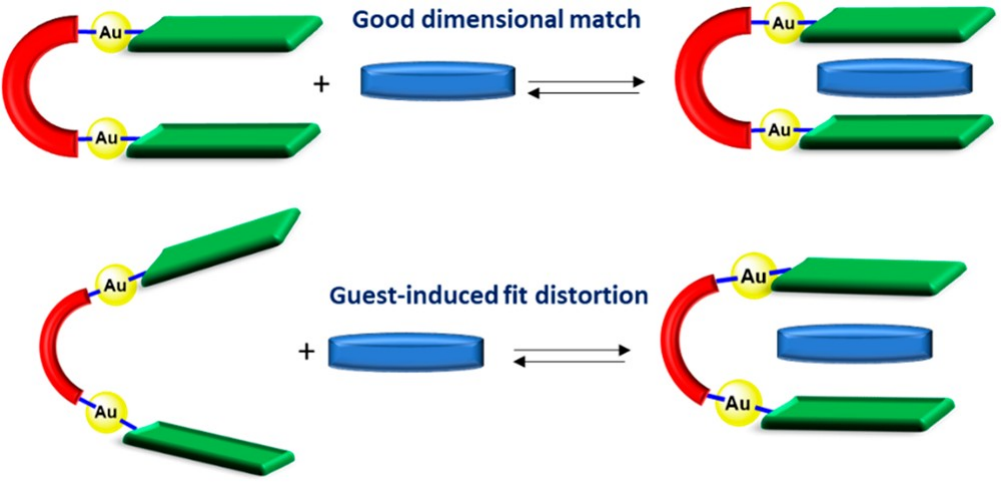

Two different metallotweezers, each with two pyrene–imidazolylidene–gold(I) arms, were used as hosts for a series of planar aromatic guests. The metallotweezer with a dibenzoacridinebis(alkynyl) spacer (**1**) orients the two pyrene–imidazolylidene–gold(I) arms in a parallel disposition, with an interpanel distance of about 7 Å. The second metallotweezer (**2**) contains a carbazolylbis(alkynyl) spacer that directs the two pyrene panels in a diverging orientation. Determination of the association constants via ^1^H NMR titrations demonstrates that the binding strength shown by **1** is significantly larger than that found by **2**, with binding affinities as large as 10^4^ M^–1^ (in CDCl_3_), for the encapsulation of *N*,*N*′-dimethylnaphthalenetetracarboxydiimide with **1**. The differences in the binding affinities are due to binding models associated with formation of the related host–guest complexes. While **1** operates via a “lock and key” model, in which the host does not suffer distortions upon formation of the inclusion complex, **2** operates via a guest-induced fit model. The large association constants shown by **1** with two planar guests were used for promotion of the template-directed synthesis of **1**, which in the absence of an external template is produced in an equimolecular mixture with its self-aggregated congener, clippane [**1**_2_]. This observation strongly suggests that the mechanically interlocked clippane is formed through a self-template-directed mechanism, while bonds are broken/formed during the synthetic protocol.

## Abstract 2

Guest-induced distortions and dimensional matching dictate the encapsulating differences of two digold(I)-based metallotweezers.

## Introduction

The term “molecular tweezer”, coined by Whitlock in 1978,^1^ refers to molecular receptors that contain two flat identical arms separated by a tether. During the last 30 years, molecular tweezers have received special attention because of their relevance at several levels. Because of their interesting properties in host–guest chemistry, molecular tweezers are now recognized as important recognition motifs with promising prospects for catalysis, biomedical, and optoelectronics applications.^2^ Molecular tweezers are often designed to trap planar aromatic molecules by sandwiching them between the two flat aromatic arms.^2a^ Therefore, for a molecular tweezer to be an effective receptor of flat aromatic guests, the separation between the two arms must be about 7 Å, which is twice the optimum distance for inclusion by effective π–π-stacking interactions. The role of the spacer is also of great importance because it is critical in the recognition process.^3^ Flexible spacers can adapt their conformation to maximize substrate binding and therefore operate through an “induced-fit” mechanism, but binding affinities are reduced because of the energy cost associated with the structural changes and the entropic loss associated with the reduction of conformational states upon guest binding. These thermodynamic costs can be compensated by using rigid spacers, which often lead to more selective and stronger binding. Whereas the majority of molecular tweezers feature purely organic architectures,^4^ metal-containing tweezers are now gaining popularity.^5^ One of the clearest advantages of the incorporation of metals into the structure of molecular tweezers is that the often-predictable coordination geometries of the metal units provide the possibility of predefining the product assembly. In addition, the introduction of metals to the structure of the tweezers endows rich electrochemistry and enhanced spectroscopic features, which may be used for the design of materials with promising prospects for sensing, emitting, light-harvesting, and photovoltaic applications.^6^

**Scheme 1:**
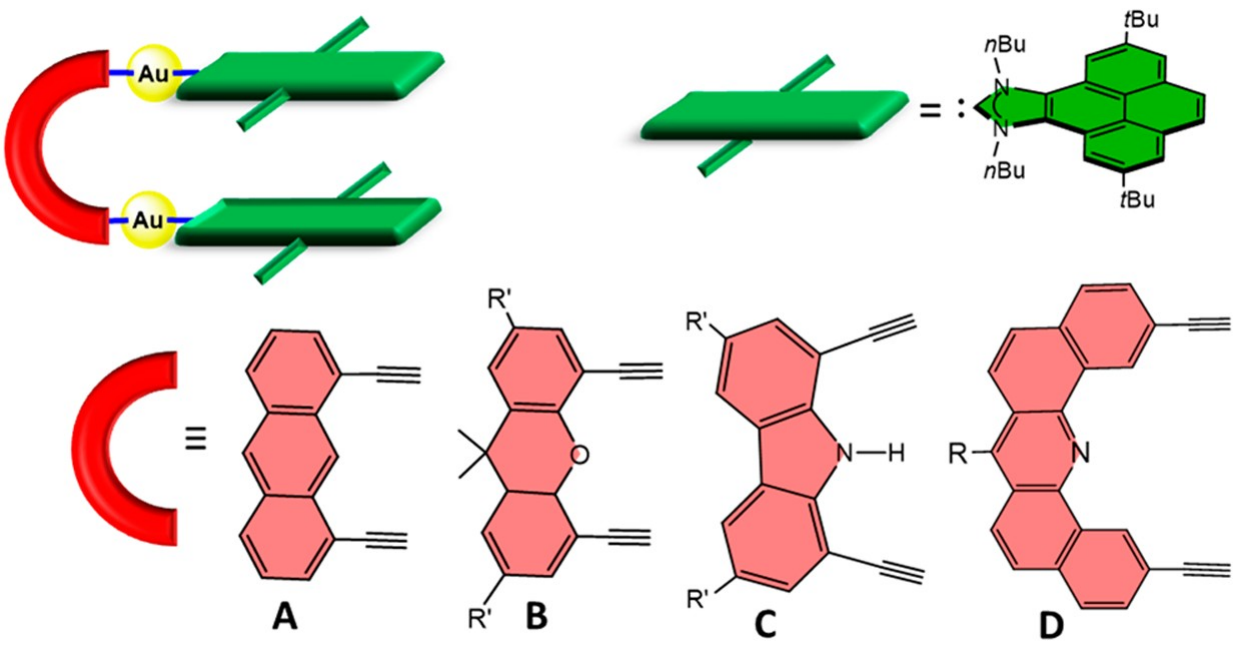
Gold(I)-Based Metallotweezers with Pyrene–Imidazolylidene–Gold(I) Arms The straight green bars across the flat green panels represent *tert*-butyl groups bound to the pyrene moieties.

During last 5 years, we contributed to the preparation of a new family of metallotweezers, formed by two Au(I)-NHC arms (NHC = N-heterocyclic carbene decorated with polyaromatic systems) linked by four different bis(alkynyl) spacers (Scheme 1). These assemblies benefit from the tendency of gold(I) complexes to show a linear geometry and from the rich chemistry of gold alkynyls, which constitute interesting tools for the construction of supramolecular assemblies.^7^ In the course of our research, we found that the supramolecular properties of our metallotweezers were greatly influenced by the nature of the spacer connecting the two flat arms of the molecule. In particular, we found that tweezers with the anthracenyl spacer **A** had a great tendency to self-aggregate, forming noncovalently bound dimers.^8^ In contrast, the complex connected with the xanthenyl spacer **B** did not self-aggregate, but the short distance between the two flat arms prevented it from being qualified as a receptor of planar aromatic substrates.^9^ Instead, this metallotweezer showed very interesting properties as a metalloligand for metals such as Cu^+^, Ag^+^, and Tl^+^. The complex with the carbazolyl spacer **C** was able to encapsulate planar aromatic guests and showed an interesting example of a guest-induced-fit conformational arrangement because the free host approaches its originally divergent arms in order to maximize the host–guest interactions.^10^ Finally, the metallotweezer with the rigid dibenzoacridine spacer **D** constituted a unique example in which both the monomer and self-aggregated structure can be formed.^11^ In this case, the dimer constituted a new type of mechanically interlocked molecule (MIM), which we named *clippane*, a term that refers to two-component MIMs formed by two fastened molecular tweezers.^11^ In the study that we report herein, we describe the ability of this metallotweezer (**1**, as numbered in Scheme 3) to encapsulate planar aromatic guests. Our decision to approach the study of the abilities of **1** as a receptor of planar guests was based on two reasons: (i) the MIM nature of [**1**_2_] eliminates the risk that the dimerization of **1** might interfere with its encapsulating abilities (**1** and [**1**_2_] are independent molecules that do not interconvert), and (ii) the structural features of **1** (rigid spacer and a distance close to 7 Å between the flat aromatic arms) make this molecule an excellent candidate for encapsulating planar molecules without suffering important guest-induced conformational distortions; hence, large host–guest affinities may be expected.

## Results and Discussion

**Scheme 2:**
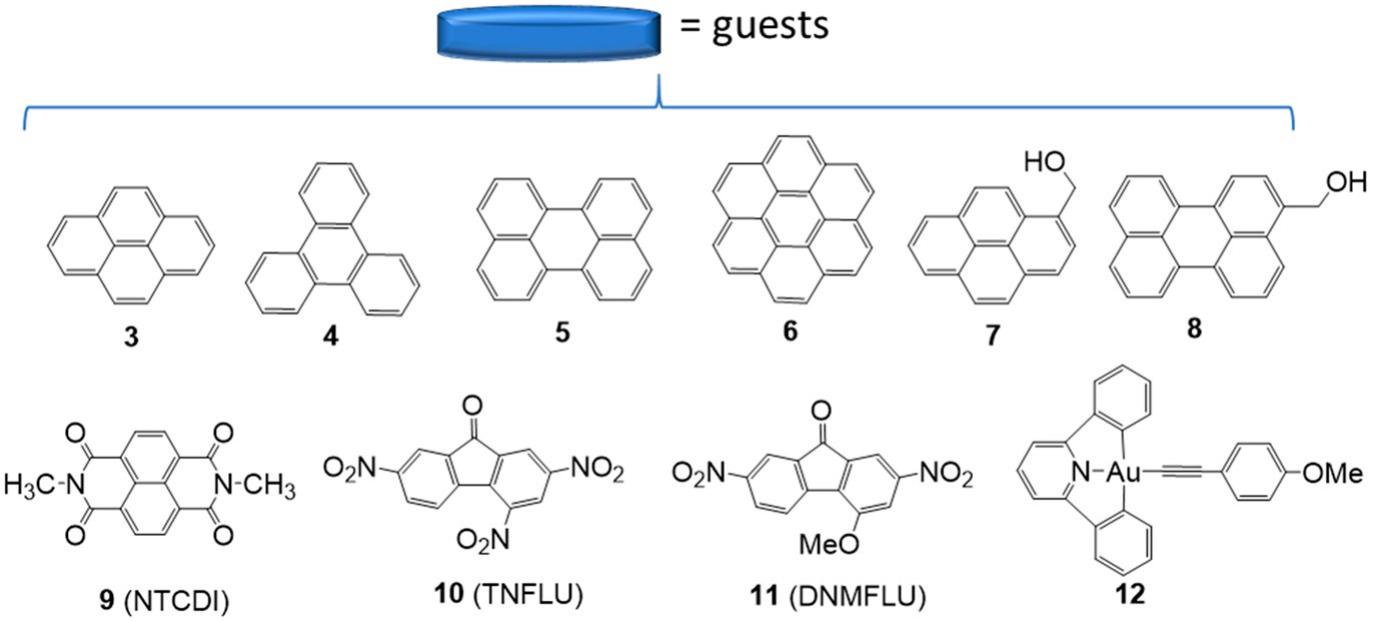
Guests Used on This Study

**Scheme 3:**
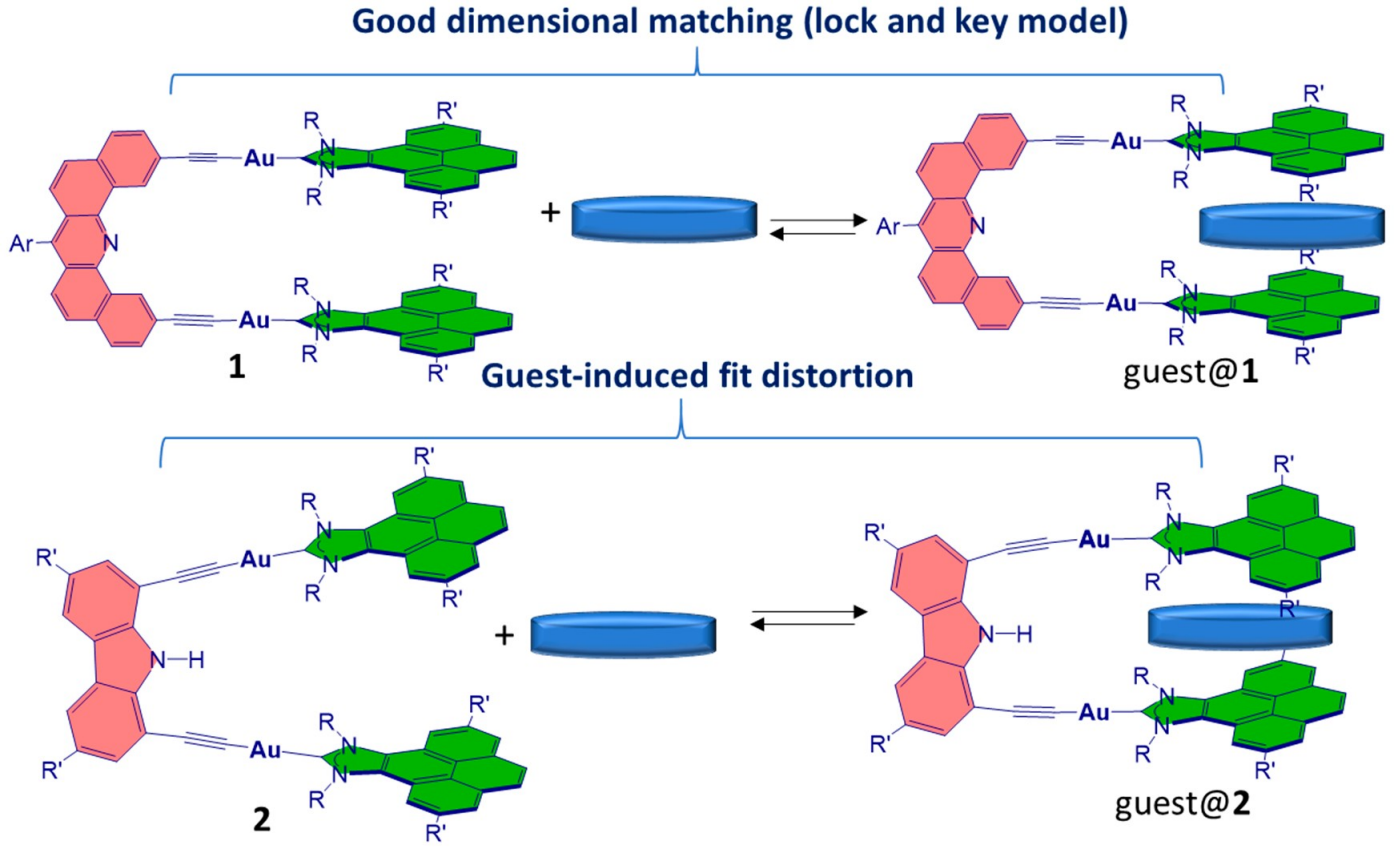
Metallotweezers Used on This Study **1** is built with linker **D**; **2** is built with linker **C**.

Scheme 2 shows the list of planar molecules that were used as guests to form the corresponding inclusion complexes with the acridine-connected metallotweezer **1**. The list includes a series of polycyclic aromatic hydrocarbons (PAHs), two methanol-functionalized PAHs, three electron-deficient planar molecules, and one pseudo-square-planar gold(III) complex with a CNC pincer ligand. The selection of these guests was performed with the aim of determining how different parameters, such as the size, the presence of hydrogen-bonding groups, or their electron-rich/poor nature, could influence binding with the metallotweezer. For comparative purposes, we also used the metallotweezer **2**, with the carbazolylbis(alkynyl) linker **C**, as the host for the same list of planar molecules. As mentioned above, given the divergent orientation of the two alkynyl groups in **2**, this metallotweezer needs to adapt its shape by approaching its two flat arms in order to maximize the face-to-face overlap with the planar guests (Scheme 3).

**Figure 1:**
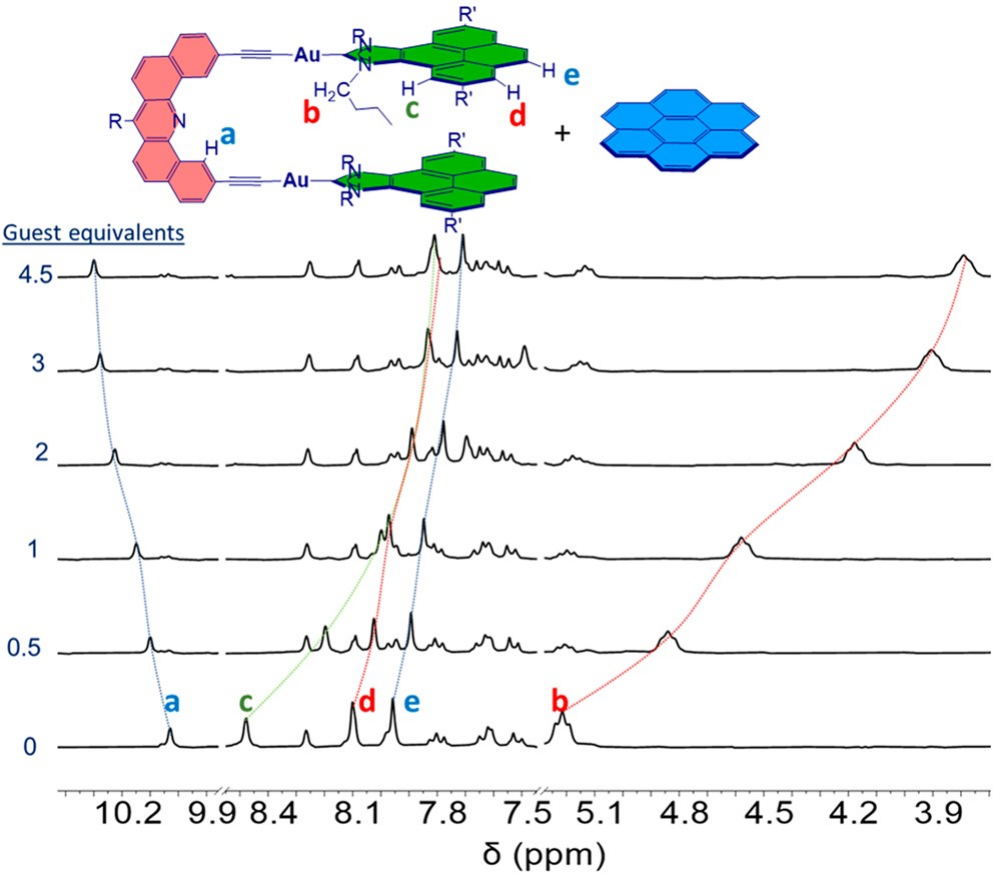
Selected region of the ^1^H NMR spectra resulting from the titration (CDCl_3_) of **1** with coronene.

In order to quantify the binding affinities of **1** and **2** with the planar guests depicted in Scheme 2, we performed ^1^H NMR titrations in CDCl_3_ at constant concentrations of the hosts. A comparison of the spectra resulting from the titrations showed that the addition of guests induced the shifting of several resonances of the protons of the hosts, an indication that formation of the inclusion complexes displays fast kinetics on the NMR time scale. In particular, the signals due to the protons of the pyrene moiety that form the flat arms of the metallotweezers and the signal due to the protons of the methylene group bound to the nitrogen atom of imidazolylidene shifted upfield with increasing concentration of the guests. In the case of the molecular tweezer **1**, the two inner C–H protons (the cove protons) of the linker are downfield-shifted upon the addition of guests, except for the titrations performed with 2,4,7-trinitro-9-fluorenone (TNFLU) and 2,7-dinitro-4-methoxyfluorenone (DNMFLU), for which these two protons are shifted upfield. This upfield shift observed for these two electron-deficient guests is an indication that the carbonyl group of fluorenene is pointing toward the spacer unit,^12^ very likely because of hydrogen-bonding interaction with the two cove protons of the spacer, which are consequently deshielded. As an example, Figure 1 shows the selected region of the ^1^H NMR spectra resulting from the titration of **1** with coronene. For this case, it can be observed that the resonance due to the cove protons of the dibenzoacridine linker is shifted downfield by +0.3 ppm, while the signals due to the protons of the N–C*H*_2_ group are shifted by −1.4 ppm. In addition, all three signals due to the protons of the pyrene moiety of the receptor are upfield-shifted by 0.3–1.2 ppm, which is a clear indication that they interact with the guest through a π–π-stacking event. On the basis of the changes observed from these titrations, the association constants with all 10 guests were determined by global-fitting analysis.^13^ Analysis of the curve fittings and a comparison of the distribution of the residuals of the 1:1 and 1:2 models allowed us to conclude that the data were best fitted to a 1:1 stoichiometry.^14^ The results shown in Table 1 indicate that the binding affinities of the nonfunctionalized PAHs (**3**–**6**) are in the order pyrene < triphenylene < perylene < coronene, as observed from the data shown in Table 1. This order, together with the relative changes in the binding constants, is consistent with the trend observed when the binding affinities of the PAH guests are compared with hosts with large portals and is a consequence of the more effective π–π-stacking overlap produced as the electron richness of the guest increases.^15^ The effect of adding a hydrogen-bonding group to the PAH molecule has a positive effect on the resulting binding constant, as can be seen for a comparison of the values obtained for pyrene and 1-pyrenylmethanol (entries 1 and 5) and perylene and 3-perylenylmethanol (entries 3 and 6). In both cases, incorporation of the hydroxyl group to the periphery of the PAH guest produces on average a 2.5-fold increase in the association constant, very likely due to the stabilization produced by the hydrogen-bonding interaction between the −OH group and the lone pair of the nitrogen atom at the acridine linker. Complex **1** is significantly effective for the encapsulation of electron-poor molecules such as *N*,*N*′-dimethylnaphthalenetetracarboxydiimide (NTCDI) and TNFLU (entries 7 and 8), with a significantly large binding constant close to 10^4^ M^–1^ shown by the first one. The replacement of one of the nitro groups in TNFLU by an electron-donating methoxy group in TNFLU to afford DNMFLU has dramatic consequences in the binding affinity of the substrate, which goes down to 227 M^–1^ (entry 9, compared with the value of 1241 M^–1^ shown by TNFLU in entry 8). The metallotweezer **1** was also able to encapsulate the pseudo-square-planar gold(III) complex **12**, showing a binding constant of 1024 M^–1^ (entry 10).

**Table 1:** Association Constants (M^–1^) Obtained for Complexation of **1** and **2** with Different Guests^a^

		*K*_1_/M^–1^	
entry	guest	**1**	**2**
1	pyrene (**3**)	74 ± 3	<10
2	triphenylene (**4**)	140 ± 3	<10
3	perylene (**5**)	373 ± 8	63 ± 1
4	coronene (**6**)	1251 ± 14	138 ± 1
5	1-pyrenylmethanol (**7**)	179 ± 5	<10
6	3-perylenylmethanol (**8**)	986 ± 35	
7	NTCDI (**9**)	9933 ± 990	1014 ± 30^b^
8	TNFLU (**10**)	1241 ± 88	762 ± 30
9	DNMFLU (**11**)	227 ± 5	170 ± 3^b^
10	Au(CNC)(C≡CC_6_H_4_-*p*-OCH_3_) (**12**)	1024 ± 24	118 ± 2^b^

a *K*_11_ values calculated by global nonlinear regression analysis.^13^ Titrations carried out by ^1^H NMR spectroscopy, using a constant concentration of the host of 0.7 mM in CDCl_3_ at 298 K. Errors refer to the nonlinear regression fittings.

b Values taken from ref (10b).

Very interesting information can be extracted from a comparison of the binding affinities of metallotweezers **1** and **2** with the planar guests shown in Scheme 1. As can be observed from the data shown in Table 1, complex **1** consistently provides larger binding affinities than complex **2** does. This is particularly relevant for the case of the encapsulation of PAH molecules (entries 1–6), the planar gold(III) complex **12** (entry 7), and the electron-poor guest NTCDI (entry 10), for which the binding affinity shown by **1** is about 1 order of magnitude larger than that shown by **2**. For the two other planar guests (NTFLU and DNMFLU), this difference is less significant. Given that the main recognition sites of both hosts **1** and **2** reside on the identical planar pyrene moieties located on the arms of the tweezers, the differences in the binding affinities should be assigned to the structural differences of these two receptors. As mentioned above, while we have an excellent shape and dimensional matching between **1** and all planar guests, the metallotweezer **2** suffers an induced-fit conformational arrangement in order to maximize the face-to-face overlap between its pyrene arms and the planar surface of the guests. This was clearly evidenced by comparing the molecular structures of the free host **2** and some of the guest@**2** host–guest complexes that we published in previous studies.^10b^ This distortion has an energy cost that renders much lower binding affinities than those observed for **1**.

**Scheme 4:**
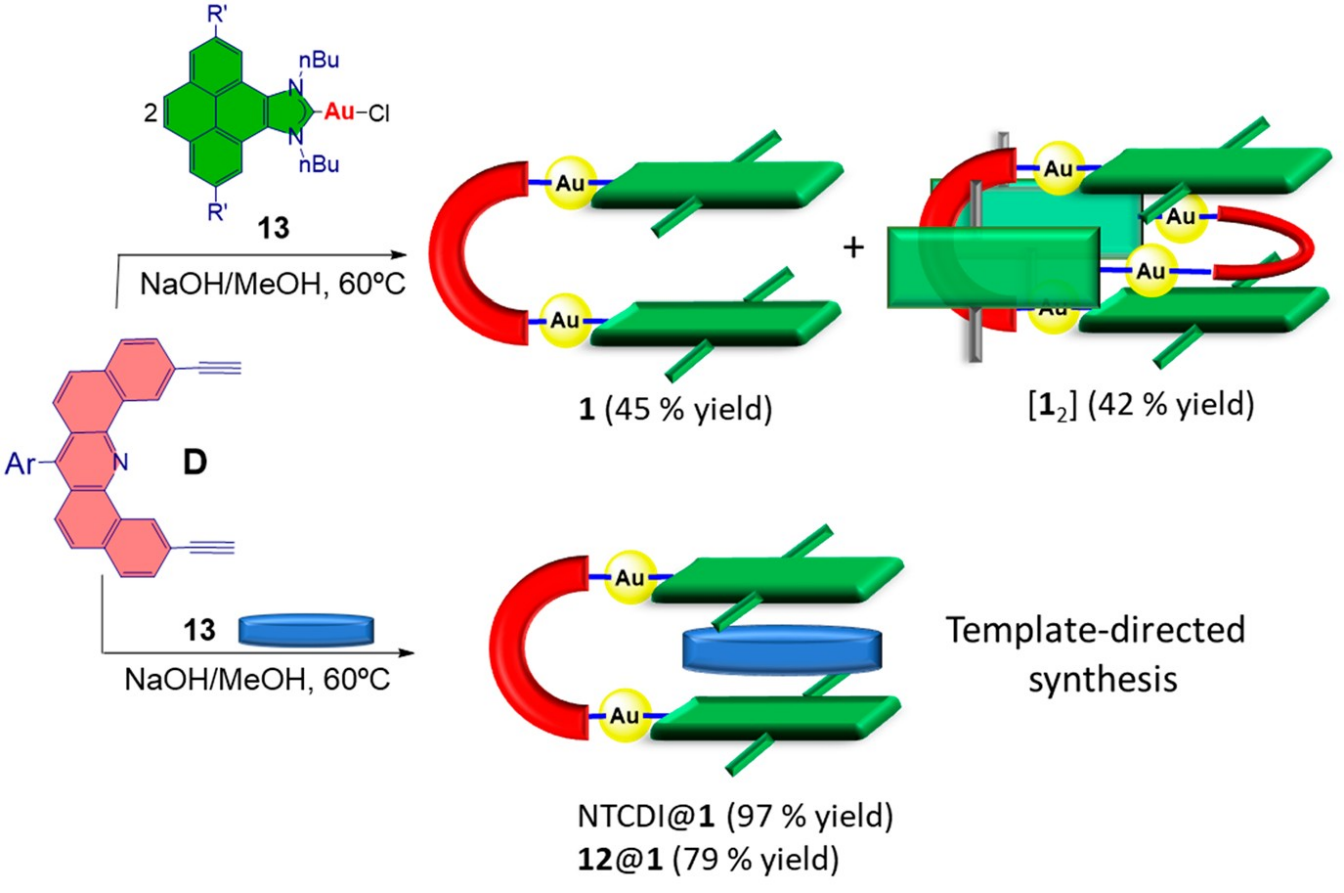
Comparison of the Products from Formation of the Metallotweezer **1** in the Presence and Absence of an External Template

Given the large association constants found for the formation of host–guest complexes between **1** and NTCDI, we wondered whether we could use this guest for a template-directed synthesis of **1**. The template effect has been used for preorganizing the reagents as a way to favor a thermodynamically controlled synthesis of supramolecular architectures.^16^ As we already reported,^11^ the preparation of the metallotweezer **1** was performed by reaction of the pyreneimidazolylidene complex **13** with 1,12-diethylnyl-[7-(3,5-di-*tert*-butylphenyl)dibenzo[*c*,*h*]acridine] (**D**) in refluxing methanol in the presence of NaOH. The reaction invariably yielded a mixture of the gold metallotweezer **1**, together with the clippane [**1**_2_] (Scheme 4), in a 1:1 molar ratio. Because of the MIM nature of [**1**_2_], these two complexes do not interconvert, and this allows them to be separated by simple column chromatography. Now we performed the reaction under the same conditions but using NTCDI as the template, and we observed that the process selectively yielded the inclusion complex of **1** with NTCDI, NTCDI@**1**, in quasi-quantitative yield. The inclusion complex NTCDI@**1** was characterized by NMR spectroscopy. The diffusion-ordered NMR spectrum showed that all of the resonances display the same diffusion coefficient, indicating that the molecule of NTCDI is associated with the molecular tweezer **1**, forming a single assembly (see the Supporting Information for details). This experiment is very interesting because one of the questions that remained unanswered when we reported the preparation of the clippane [**1**_2_] was, how was this MIM formed? Given that **1** and [**1**_2_] do not interconvert even at high temperatures and during long periods of time, we assumed that [**1**_2_] was formed during the synthetic process, very likely as a consequence of a self-template effect. The fact that the synthesis of [**1**_2_] can be inhibited in the presence of a template is clear proof of this assumption because the use of an external template avoids the possibility that self-templation directs the synthesis. In order to see whether a guest with lower binding affinities could also facilitate the template-directed synthesis of **1**, we used the gold(III) complex **12** as a template and observed that the inclusion complex **12**@**1** was formed in 79%. Under these reaction conditions, we did not observe formation of the clippane [**1**_2_].

## Conclusions

In this work, we showed the excellent encapsulating properties of a digold metallotweezer toward a large series of planar guests. The receptor benefited from the presence of a rigid dibenzoacridinebis(alkynyl) linker, which allowed a parallel orientation of the two pyridine–imidazolylidene–gold(I) panels at a distance of about 7 Å.^3b,17^ The large binding affinities of this receptor contrast with the much lower ones shown by a similar metallotweezer with a carbazolylbis(alkynyl) linker, which must change its structural configuration in order to maximize the π–π interaction with the guests, thus paying an energy cost that justifies the lower association constants. In other words, the energy cost required by the carbazolyl-linked metallotweezer **2** to force the unparallel arms to become parallel in the host–guest complex is the main factor that justifies the lower encapsulating abilities of **2**. The study illustrates how subtle differences in the geometry of molecular receptors may have dramatic effects on the encapsulating properties of the systems. In the particular examples that we describe in this study, we present two systems that reflect the transition between a host–guest “lock and key” model and an “induced-fit” model. The “lock and key” model was established by Fischer in 1894^18^ and reflects how the substrate fits into the receptor like a key in a lock. Hosts operating through the “lock and key” model minimize the entropic cost of conformational selection and, consequently, increase their binding abilities.^3a^ In our case, this lock and key behavior does not just refer to a purely geometrical fit because the host molecule possesses complementary groups that enhance its binding abilities and offers a directional orientation of the guest within the cavity of the host, as shown for the guests with hydrogen-bond donors (pyrenylmethanol and perylenylmethanol) and hydrogen-bond acceptors (TNFLU and DNMFLU).

We took advantage of the large binding affinities shown by the molecular tweezer **1** with two planar guests to promote its template-directed synthesis. When the reaction was carried out in the absence of a guest template, an equimolecular mixture of **1** and the clippane [**1**_2_] was formed. When the same reaction was carried out in the presence of NTCDI or the planar gold(III) complex **12**, the reaction was directed toward the inclusion complexes NTCDI@**1** and **12**@**1** and the clippane is not observed. This result is of particular relevance because it sheds light on the process of formation of the clippane [**1**_2_], which is very likely formed by a self-template-directed process, which is inhibited when a strong π–π-stacking binder is added to the reaction vessel. The mechanism of formation of [**1**_2_], which remained elusive in our previous studies, could give us new hints for the preparation of further clippanes or metallotweezer-derived MIMs.

## Supporting Information Available

Preparation and characterization of the new host–guest species, determination of the association constants, and NMR and mass spectra (PDF)
