# Supplementary material for: “Lock and Key” and “Induced-Fit” Host–Guest Models in Two Digold(I)-Based Metallotweezers
Source: Inorg Chem. 2022 Apr 1;62(5):1820–6. doi: 10.1021/acs.inorgchem.2c00677 (PMC9974064; doi:10.1021/acs.inorgchem.2c00677)
Supplement: Supplementary file 1 — ic2c00677_si_001.pdf [file ic2c00677_si_001.pdf]

Supporting information for:

'Lock and key' and 'induced-fit' host-guest models in two di-gold(I)-based metallotweezers

Susana Ibáñez[a]* and *Eduardo Peris*[a]*

[a] Institute of Advanced Materials (INAM). Centro de Innovación en Química Avanzada (ORFEO-CINQA). Universitat Jaume I. Av. Vicente Sos Baynat s/n. Castellón. E-12071. Spain. Email: maella@uji.es, eperis@uji.es

General considerations **S2**

1. Synthesis of the complexes **S2**

2. Spectroscopic data **S5**

2.1. ^{1}H and ^{13}C NMR spectra of **NTCDI@1** S5

2.2. ^{1}H and ^{13}C NMR spectra of **12@1** S6

3. Titration experiments **S7-S26**

3.1. ^{1}H NMR titrations **S7**

4. DOSY experiment **S27**

5. References **S28**

General considerations. The pyrene-imidazolylidene-gold(I) complex **13**,[1] 1,12-diethylnyl-[7-(3,5-di-*tert*-butylphenyl)dibenzo[*c,h*]acridine],[2] N,N'-dimethyl-naphthalenetetracarboxy diimide (NTCDI),[3] 2,7-dinitro-4-methoxy-fluorenone (DNMFLU),[4] Au(C^N^C)(C≡CC_6H_4-OCH_3-*p*) (**12**),[5] carbazolyl-bis-alkynyl di-gold(I) metallotweezer (**2**)[6] were prepared according to literature methods. All other reagents were used as received from commercial suppliers. Infrared spectra (FTIR) were performed on a FT/IR-6200 (Jasco) spectrometer equipped with a Pro One ATR with a spectral window of 4000-400 cm^{-1}. Elemental analyses were carried out on a TruSpec Micro Series. NMR spectra was recorded on a Bruker 400 MHz or Bruker 300 MHz using $CDCl_3$ as solvents. ESI and ESI IM mass spectra were performed using a SYNAPT XS High-Definition Mass Spectrometer (Waters Corporation, Manchester, UK) equipped with an electrospray ionization (ESI) source. The ions generated are transmitted through the StepWave XS ion guide to the first quadrupole (Q), then to the traveling wave ion mobility (TWIM) cell, and finally analyzed with a time-of flight (TOF) mass analyzer.

1. Synthesis of the complexes

Synthesis of complex NTCDI@1. 1,12-diethylnyl-[7-(3,5-di-*tert*-butylphenyl)dibenzo[*c,h*]acridine] (37.4 mg, 0.072 mmol) and NaOH (14.50 mg, 0.363 mmol) were dissolved in deoxygenated methanol (20 mL). The solution was heated at reflux for 1 hour. Then, complex **13** (101.28 mg, 0.145 mmol) and N,N'- dimethyl-naphthalenetetracarboxy diimide, (NTCDI) (42.68 mg, 0.145 mmol) was added as a solid and the resulting suspension was stirred for 18 h at 60°C. The mixture was evaporated to dryness and the solid residue was extracted with dichloromethane, and the solution was filtered through a short pad of Celite. Complex **NTCDI@1** was isolated as a red solid. Yield: 153.7 mg, 98%. IR (ATR): ν(C≡C): 2111.67 cm^{-1} Electrospray ESI-MS (*m/z*): 2158.0 [**NTCDI@1**+Na]$^+$. Anal. Calcd. for $C_{121}H_{125}N_7O_4Au_2$ (2135.22): C, 68.06; H, 5.90; N, 4.59. Found: C, 68.08; H, 5.85; N, 4.80. ^{1}H NMR (400 MHz, $CDCl_3$): δ 10.12 (s, 2H, C*H*), 8.64 (d, 2H, $^3J_{H-H}$ = 12.0 Hz, C*H*), 8.25 (s, 1H, C*H*), 8.03 (s, 4H, CH_{pyr}), 7.94 (d, 2H, $^3J_{H-H}$ = 8.0 Hz, C*H*), 7.78 (d, 2H, $^3J_{H-H}$ = 8.0 Hz, C*H*), 7.72 (s, 4H, CH_{pyr}), 7.68-7.49 (m, 8H, CH_{pyr} + CH_{NTCDI}), 7.41 (s, 2H, C*H*), 7.33 (s, 2H, C*H*), 5.02-4.85 (m, 8H, $NCH_2CH_2CH_2CH_3$), 3.61 (s, 6H, $CH_{3\ NTCDI}$), 2.29-1.87 (m, 8H, $NCH_2CH_2CH_2CH_3$), 1.74-1.54 (m, 8H, $NCH_2CH_2CH_2CH_3$), 1.53 (s, 36H, $C(CH_3)_{3\ pyr}$), 1.45 (s, 18H, $C(CH_3)_3$), 1.13-0.95 (m, 12H, $NCH_2CH_2CH_2CH_3$). ^{13}C {^{1}H} NMR (100 MHz, $CDCl_3$): δ 193.36 (Au-$C_{carbene}$), 162.24 ($C_{q\ C≡O\ NTCDM}$), 150.90 (C_q), 149.29 (C_q), 149.04 ($C_{q\ pyr}$), 145.54 (C_q

$_{pyr}$), 132.99 (*C*H), 132.09 (*C*$_{q\ pyr}$), 130.75 (*C*$_{q\ NTCDI}$), 130.06 (*C*$_{q\ pyr}$), 129.87 (*C*H), 128.02 (*C*H$_{pyr}$), 128.02 (*C*$_q$), 127.24 (*C*$_{q\ pyr}$), 127.56 (*C*H), 127.03 (*C*H), 125.18 (*C*H), 124.50 (C$_q$), 124.12 (*C*$_{q\ NTCDI}$), 124.01 (*C*H$_{NTCDI}$), 123.22 (C$_q$), 123.01 (*C*H), 122.50 (*C*H$_{pyr}$), 121.66(C$_q$), 119.83 (*C*$_{q\ acetylide}$), 119.49 (*C*$_{q\ acetylide}$), 116.83 (*C*H), 116.60 (*C*H$_{pyr}$), 106.32 (*C*$_q$), 52.08 (N*C*H$_2$CH$_2$CH$_2$CH$_3$), 35.40 (*C*(CH$_3$)$_{3pyr}$), 35.19 (*C*(CH$_3$)$_3$), 32.73 (NCH$_2$*C*H$_2$CH$_2$CH$_3$), 31.90 (C(*C*H$_3$)$_{3pyr}$), 31.73 (C(*C*H$_3$)$_3$), 27.96 (*C*H$_{3NTCDM}$), 20.36 (NCH$_2$CH$_2$*C*H$_2$CH$_3$), 14.09 (NCH$_2$CH$_2$CH$_2$*C*H$_3$).

Synthesis of complex 12@1. 1,12-diethylnyl-[7-(3,5-di-tert-butylphenyl)dibenzo[c,h]acridine] (30.00 mg, 0.057 mmol) and NaOH (11.36 mg, 0.284 mmol) were dissolved in deoxygenated methanol (20 mL). The solution was heated at reflux for 1 hour. Then, complex **13** (79.34 mg, 0.114 mmol) and Au(C^N^C)(C≡CC$_6$H$_4$-OCH$_3$-p)(**3**) (31.66 mg, 0.057 mmol) was added as a solid and the resulting suspension was stirred for 18 h at 60°C. The mixture was evaporated to dryness and the solid residue was extracted with dichloromethane, and the solution was filtered through a short pad of Celite. Complex **12@1** was isolated as a yellow solid. Yield: 107.2 mg, 79%. IR (ATR): ν(C≡C): 2145.42 and 2105.89 cm^{-1}. Electrospray ESI-MS (m/z): 2399.0 [**3@2**+H]$^+$. Anal. Calcd. for C$_{131}$H$_{133}$N$_6$OAu$_3$ (2140.89): C, 65.60; H, 5.59; N, 3.50. Found: C, 65.68; H, 5.52; N, 3.52. ^{1}H NMR (300 MHz, CDCl$_3$): δ 10.16 (s, 2H, CH), 8.65 (d, 2H, $^3J_{H-H}$ = 12.0 Hz, C*H*), 8.26 (s, 1H, C*H*), 8.19 (s, 4H, C*H*$_{pyr}$), 8.08 (d, 2H, $^3J_{H-H}$ = 6.0 Hz, C*H*), 7.95 (d, 2H, $^3J_{H-H}$ = 6.0 Hz, C*H*), 7.81 (d, $^3J_{H-H}$ = 8.0 Hz, 2H, C*H*$_{Au(III)}$), 7.70 (s, 4H, C*H*$_{pyr}$), 7.66 (s, 2H, C*H*), 7.64 (s, 4H, C*H*$_{pyr}$), 7.54 (s, 2H, C*H*), 7.36 (m, 2H, C*H*$_{Au(III)}$), 7.21 (t, $^3J_{H-H}$ = 15 Hz, $^3J_{H-H}$ = 9.0 Hz, 3H, C*H*$_{Au(III)}$), 7.13 (t, $^3J_{H-H}$ = 8.0 Hz, $^3J_{H-H}$ = 6.0 Hz, 2H, C*H*$_{Au(III)}$), 6.86 (d, $^3J_{H-H}$ = 9 Hz, 2H, C*H*$_{Au(III)}$), 6.70 (d, $^3J_{H-H}$ = 9 Hz, 2H, C*H*$_{Au(III)}$), 4.95-4.76 (m, 8H, NC*H*$_2$CH$_2$CH$_2$CH$_3$), 3.08 (s, 3H, OC*H*$_{3Au(III)}$), 2.04-1.85 (m, 8H, NCH$_2$C*H*$_2$CH$_2$CH$_3$), 1.80-1.67 (m, 8H, NCH$_2$CH$_2$C*H*$_2$CH$_3$), 1.44 (s, 36H, C(C*H*$_3$)$_{3\ pyr}$), 1.42 (s, 18H, C(C*H*$_3$)$_3$), 0.92 (t, $^3J_{H-H}$= 4.0 Hz, 12H, NCH$_2$CH$_2$CH$_2$C*H*$_3$). ^{13}C {^{1}H} NMR (100 MHz, CDCl$_3$): δ 192.86 (Au-*C*$_{carbene}$), 166.23 (*C*$_{q\ Au(III)}$), 163.28 (*C*$_{q\ Au(III)}$), 158.66 (*C*$_{q\ Au(III)}$), 150.89 (*C*$_{q\ Au(III)}$), 148.35 (*C*$_{q\ pyr}$), 148.29 (*C*$_{q\ pyr}$), 145.53 (*C*$_q$), 140.52 (*C*$_{q\ Au(III}$), 136.31 (*C*H), 133.125 (*C*H$_{Au(III)}$), 133.13 (*C*H), 132.23 (*C*$_q$), 132.08 (*C*H$_{Au(III)}$), 132.01 (*C*$_q$),131.37 (*C*$_{q\ pyr}$), 129.77 (*C*H), 128.50 (*C*H), 127.86 (*C*H), 127.38 (*C*H$_{pyr}$), 126.35 (*C*H$_{Au(III)}$), 125.21 (*C*$_{q\ pyr}$), 124.80 (*C*$_q$), 124.37 (*C*H$_{Au(III)}$), 124.09 (C$_{q\ pyr}$), 122.24 (*C*H$_{pyr}$), 120.98 (*C*$_{q\ acetylide}$), 120.49 (*C*$_{q\ acetylide}$), 119.07 (*C*$_{q\ acetylideAu(III)}$), 116.77 (*C*H$_{pyr}$), 115.62 (*C*H$_{Au(III)}$), 113.73 (*C*H$_{Au(III)}$), 106.18 (*C*$_q$), 100.23 (*C*$_{q\ acetylideAu(III)}$), 55.48 (O*C*H$_3$), 51.93

($NCH_2CH_2CH_2CH_3$), 35.31 ($C(CH_3)_{3pyr}$), 35.20 ($C(CH_3)_3$), 32.77 ($NCH_2CH_2CH_2CH_3$), 31.89 ($C(CH_3)_{3pyr}$), 31.73 ($C(CH_3)_3$), 20.29 ($NCH_2CH_2CH_2CH_3$), 14.11 ($NCH_2CH_2CH_2CH_3$).

2. Spectroscopic data

2.1. ^{1}H and ^{13}C NMR spectra of NTCDI@1 in $CDCl_3$

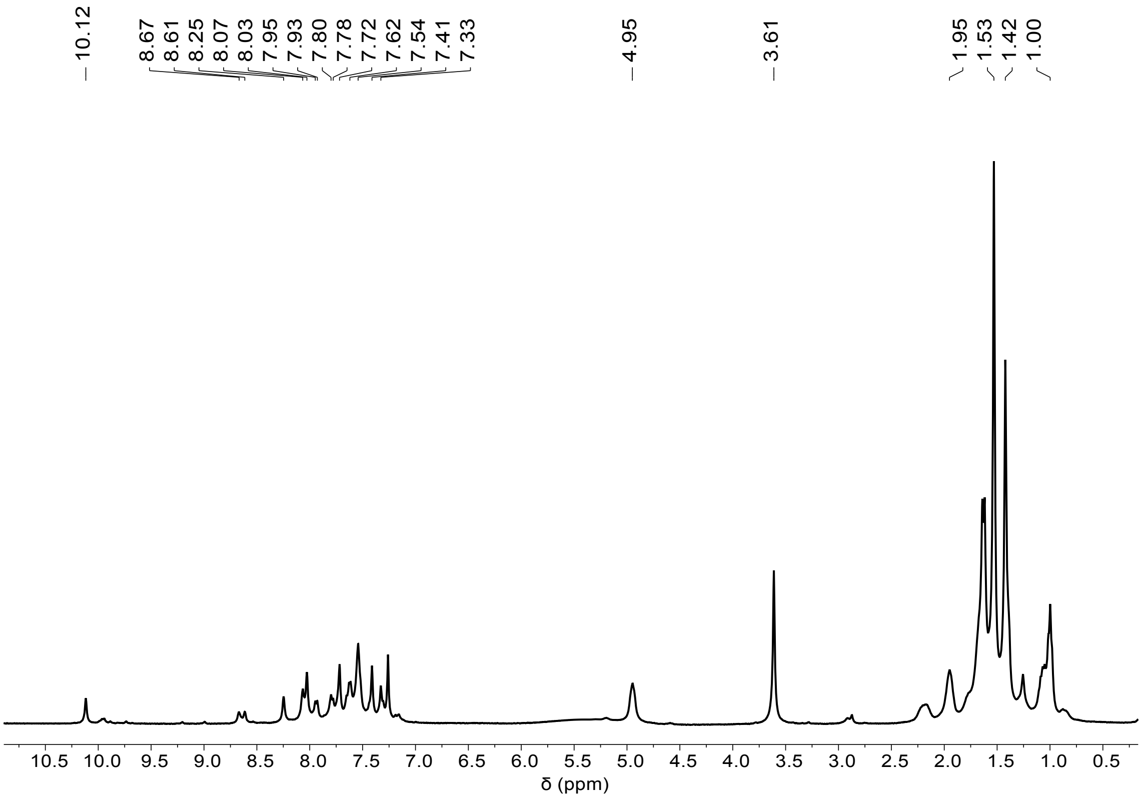

Figure S1. ^{1}H NMR spectrum of **NTCDI@1** in $CDCl_3$

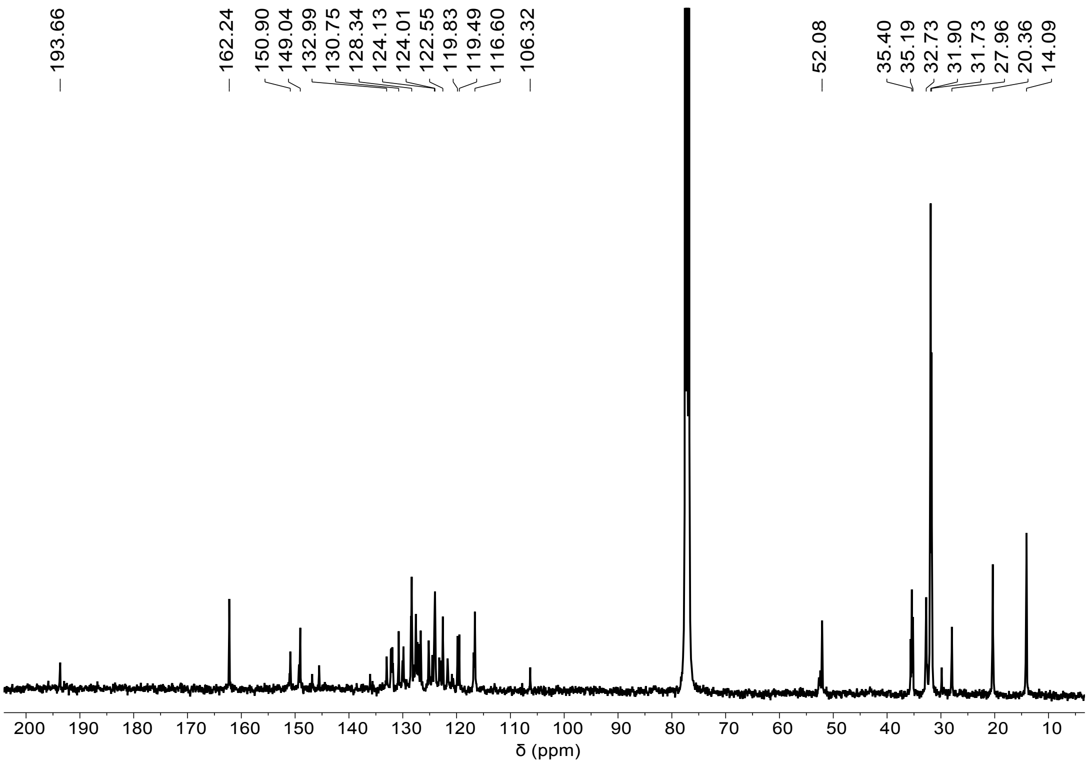

Figure S2. ^{13}C NMR spectrum of **NTCDI@1** in $CDCl_3$

2.2. ^{1}H and ^{13}C NMR spectra of 12@1 in $CDCl_3$

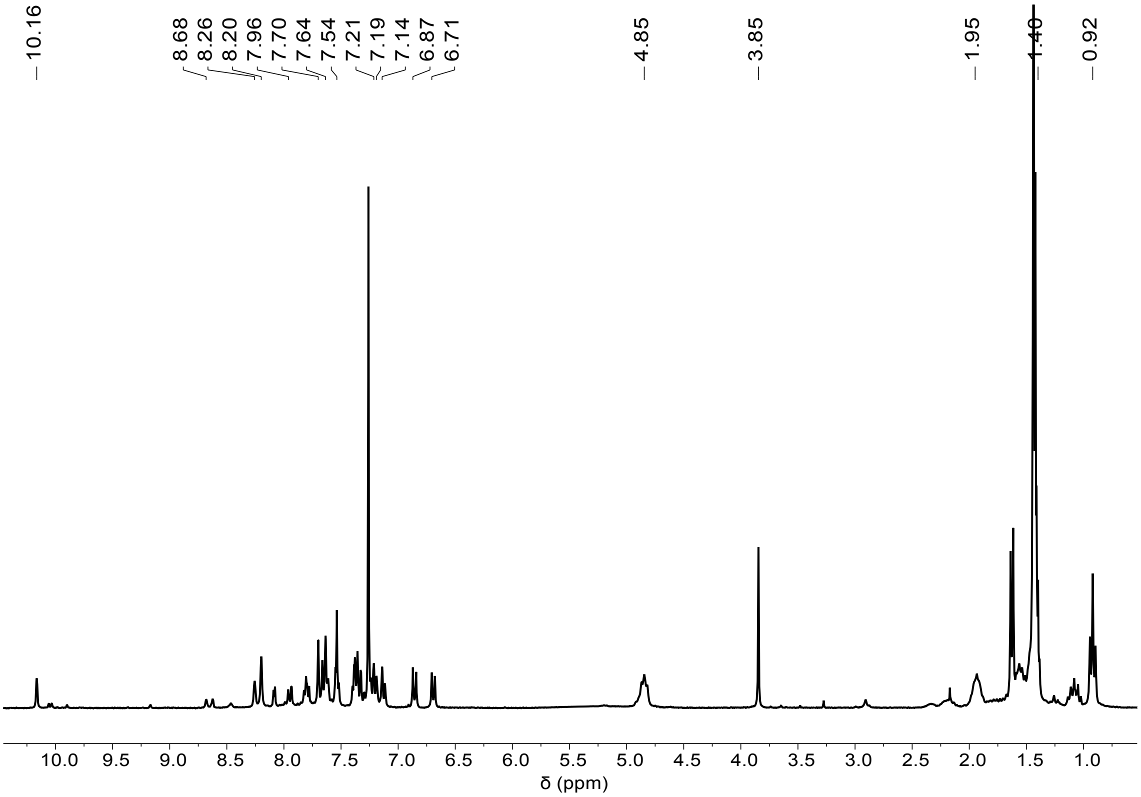

Figure S3. ^{1}H NMR spectrum of **12@1** in $CDCl_3$

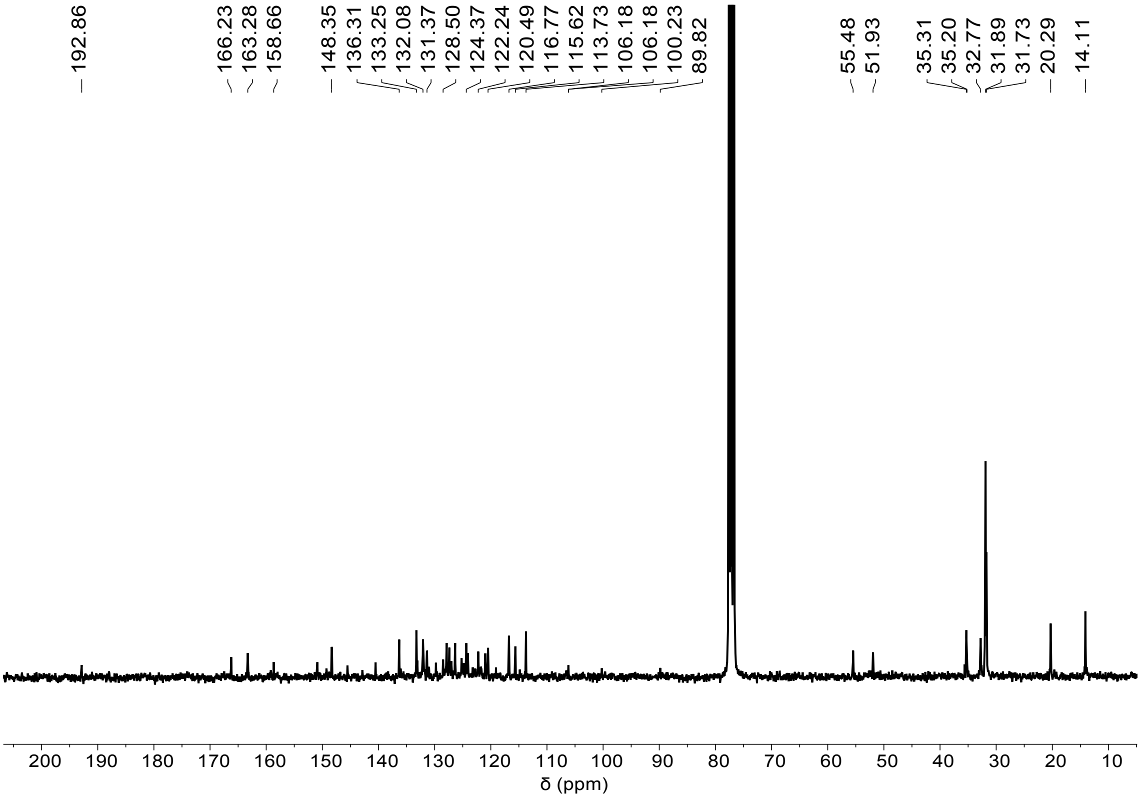

Figure S4. ^{13}C NMR spectrum of **12@1** in $CDCl_3$

3. ^{1}H NMR titration experiments

3.1. ^{1}H NMR titrations

The recognition capability of complexes **1** and **2** (host) were studied by ^{1}H NMR titration experiments, by adding increasing amounts of different electron rich organic substrates as pyrene, 1-pyrenyl-methanol, triphenylene, perylene, 3-perylenyl-methanol, coronene and poor organic substrates as N,N'-dimethyl-naphthalenetetracarboxy diimide (NTCDI), 2,4,7-trinitro-fluorenone (TNFLU), 2,7-dinitro-4-methoxy-fluorenone (DNMFLU) and (Au(C^N^C)(C≡CC$_6$H$_4$-OCH$_3$-*p*)(**12**) to a solution of complexes **1** and **2**. The experiment was carried out in $CDCl_3$, at constant concentrations of the host (0.75 mM). Two solutions were prepared: solution A (only containing host at 0.75 mM) and solution B (containing host at 0.75 mM and guest at different mM). The addition of increasing amounts of solution B to solution A produced perturbations on the signal due to the proton of the pyrene core of the host. The association constants were calculated by nonlinear least-square analysis, by using the BindFitv0.5 program.

Titration of 1 with pyrene

Table S1. Data values from the titration study of **1** with pyrene

**[1]** M	[pyrene] M	δc_H	δc_H	δc_H	δ_{NCH2}	equiv. pyrene
0,00079898	0	10,03	8,48	7,96	5,22	0
0,00079898	0,00091132	10,04	8,45	7,95	5,19	1,1
0,00079898	0,00178758	10,04	8,42	7,95	5,16	2,2
0,00079898	0,00263078	10,05	8,4	7,94	5,12	3,3
0,00079898	0,00344275	10,06	8,37	7,93	5,1	4,3
0,00079898	0,00497969	10,07	8,34	7,92	5,06	6,2
0,00079898	0,00774619	10,08	8,28	7,91	5	9,7
0,00079898	0,01016687	10,1	8,24	7,9	4,96	12,7
0,00079898	0,01420135	10,11	8,19	7,88	4,88	17,8
0,00079898	0,01742892	10,13	8,19	7,88	4,84	21,8
0,00079898	0,02323857	10,13	8,19	7,87	4,81	29,1

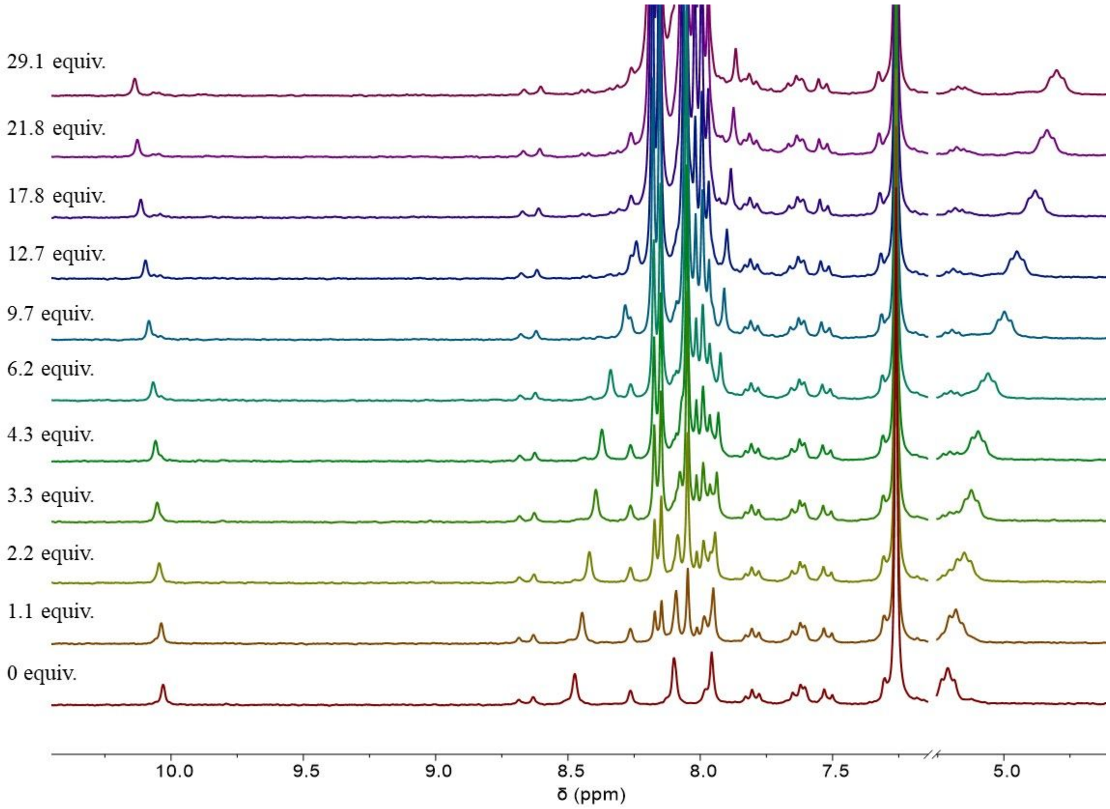

Figure S5. Selected region and spectra of the titration of complex **1** with pyrene.

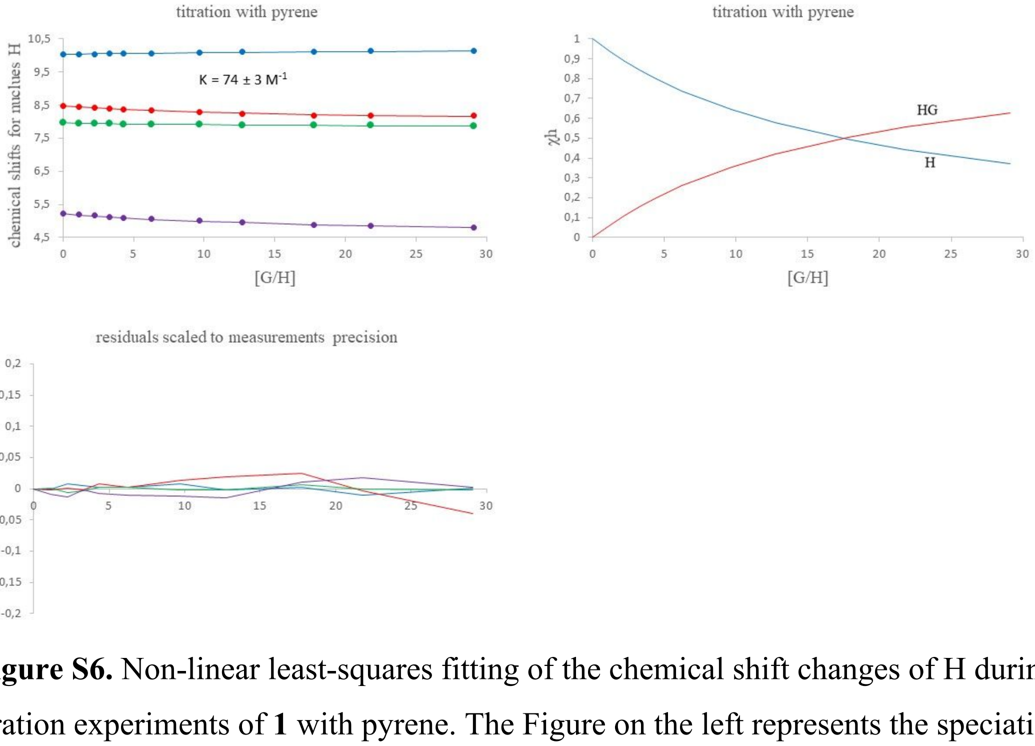

Figure S6. Non-linear least-squares fitting of the chemical shift changes of H during titration experiments of **1** with pyrene. The Figure on the left represents the speciation profiles.

Titration of 1 with 1-pyrenyl-methanol

Table S2. Data values from the titration study of **1** with 1-pyrenyl-methanol

**[1]** M	[1-pyrenyl-methanol] M	δc_H	δc_H	δ_{NCH2}	equiv. 1-pyrenyl-methanol
0,00089409	0	10,03	7,96	5,21	0
0,00089409	0,00092856	10,06	7,94	5,09	1,0
0,00089409	0,00182141	10,08	7,93	4,98	2,0
0,00089409	0,00268056	10,1	7,92	4,9	3,0
0,00089409	0,0035079	10,12	7,91	4,84	3,9
0,00089409	0,00507393	10,13	7,9	4,76	5,7
0,00089409	0,00789277	10,15	7,88	4,68	8,8
0,00089409	0,01035926	10,16	7,87	4,6	11,6
0,00089409	0,01447008	10,17	7,86	4,51	16,2
0,00089409	0,01775874	10,19	7,85	4,45	19,9
0,00089409	0,02367832	10,19	7,85	4,41	26,5

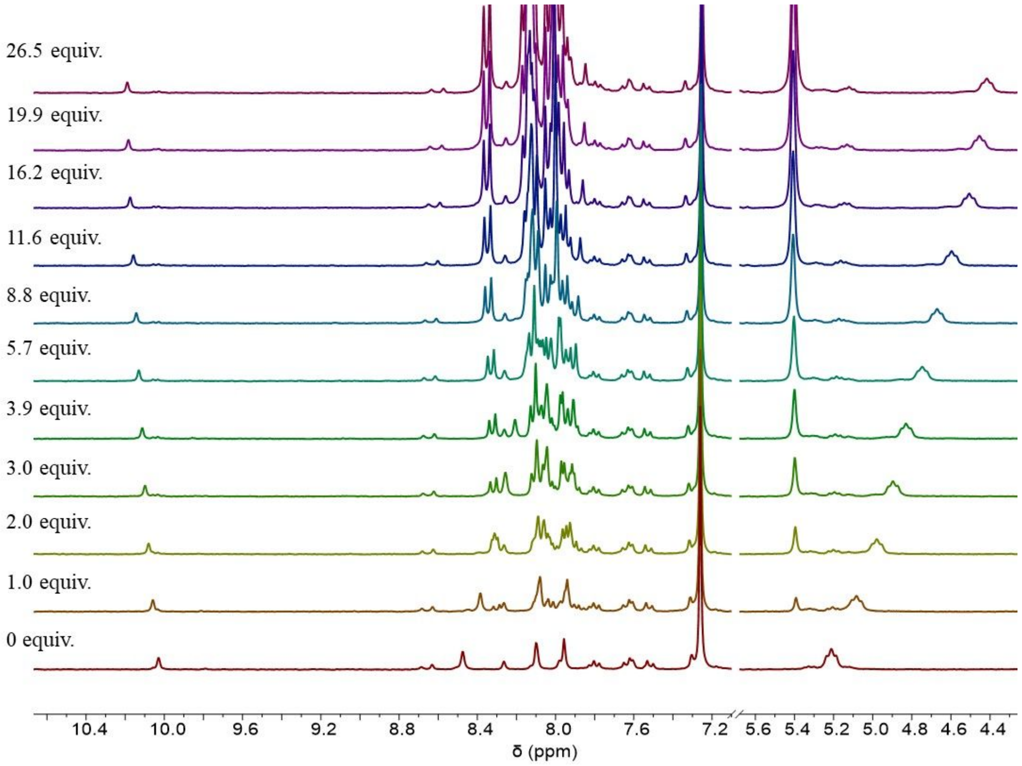

Figure S7. Selected region and spectra of the titration of complex **1** with 1-pyrenyl-methanol.

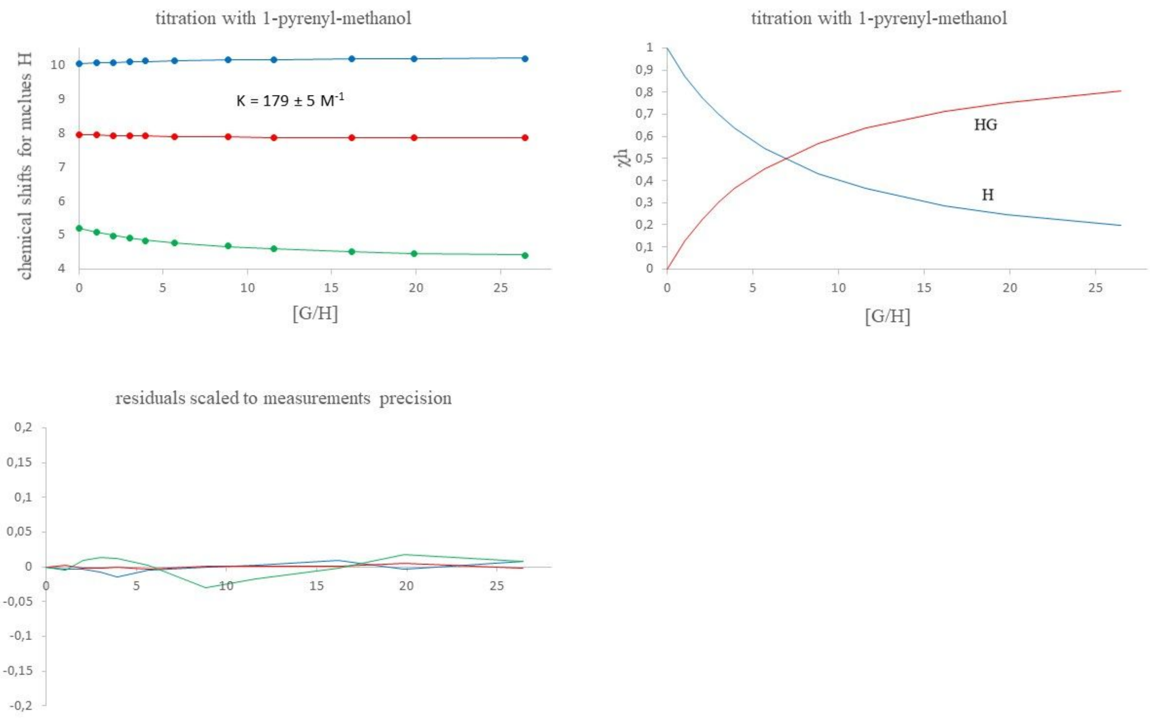

Figure S8. Non-linear least-squares fitting of the chemical shift changes of H during titration experiments of **1** with 1-pyrenyl-methanol. The Figure on the left represents the speciation profiles.

Titration of 1 with triphenylene

Table S3. Data values from the titration study of **1** with triphenylene

[**1**] M	[triphenylene] M	δc_H	δc_H	δc_H	δc_H	δ_{NCH2}	equiv. triphenylene
0,00077995	0	10,03	8,48	8,1	7,96	5,22	0
0,00077995	0,00089322	10,05	8,4	8,08	7,93	5,11	1,1
0,00077995	0,00175208	10,07	8,34	8,05	7,91	5,01	2,2
0,00077995	0,00257853	10,09	8,29	8,04	7,89	4,94	3,3
0,00077995	0,00337438	10,1	8,25	8,02	7,87	4,87	4,3
0,00077995	0,0048808	10,13	8,19	8,01	7,85	4,78	6,3
0,00077995	0,00759235	10,15	8,1	7,98	7,82	4,66	9,7
0,00077995	0,00996496	10,17	8,05	7,96	7,8	4,58	12,8
0,00077995	0,01391931	10,19	7,98	7,94	7,77	4,48	17,8
0,00077995	0,01708279	10,21	7,91	7,93	7,76	4,42	21,9
0,00077995	0,02277705	10,22	7,94	7,91	7,75	4,38	29,2

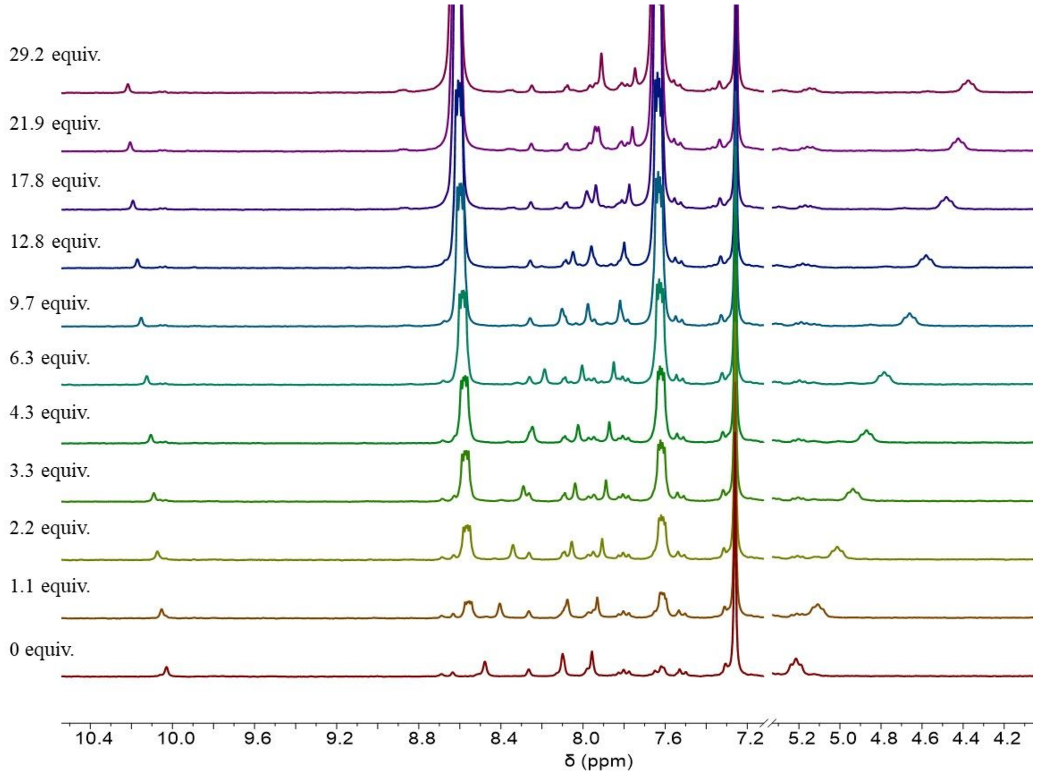

Figure S9. Selected region and spectra of the titration of complex **1** with triphenylene.

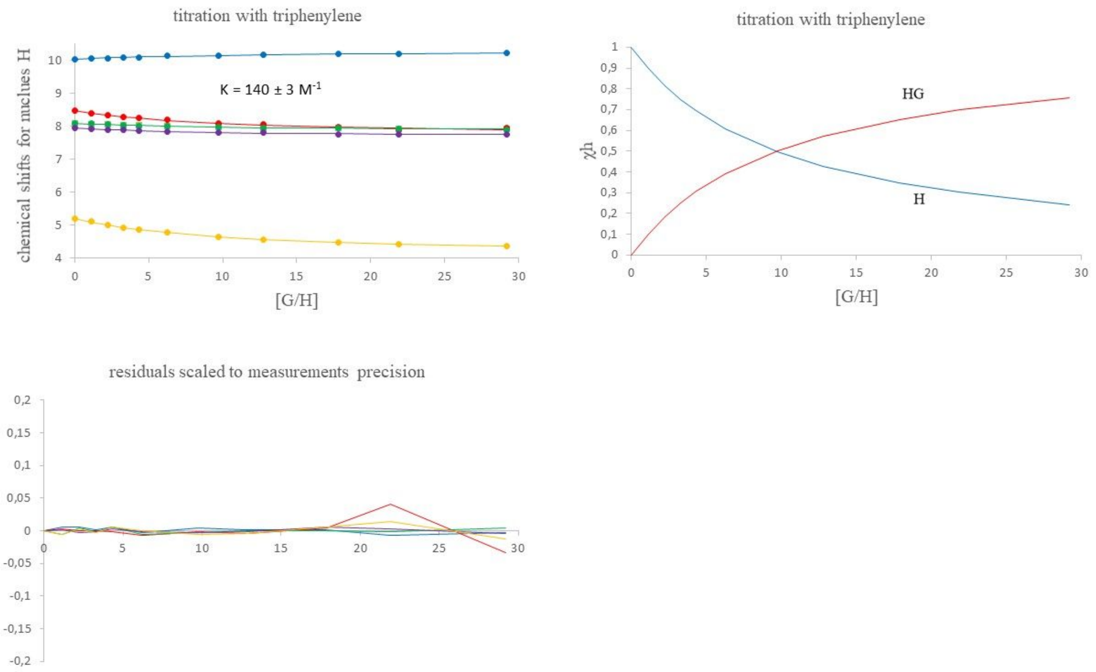

Figure S10. Non-linear least-squares fitting of the chemical shift changes of H during titration experiments of **1** with triphenylene. The Figure on the left represents the speciation profiles.

Titration of 1 with perylene

Table S4. Data values from the titration study of **1** with perylene

**[1]** M	[perylene] M	δc_H	δc_H	δc_H	δc_H	δ_{NCH2}	equiv. perylene
0,00079898	0	10,03	8,48	8,1	7,96	5,21	0
0,00079898	0,00022293	10,04	8,44	8,09	7,94	5,16	0,3
0,00079898	0,00042935	10,05	8,4	8,07	7,93	5,12	0,5
0,00079898	0,00062102	10,06	8,37	8,06	7,91	5,07	0,8
0,00079898	0,00079948	10,07	8,35	8,05	7,9	5,03	1,0
0,00079898	0,00112185	10,08	8,3	8,03	7,88	4,97	1,4
0,00079898	0,00165606	10,1	8,23	8	7,85	4,89	2,1
0,00079898	0,00208069	10,11	8,19	7,99	7,83	4,83	2,6
0,00079898	0,00271312	10,12	8,14	7,97	7,81	4,76	3,4
0,00079898	0,00316157	10,13	8,13	7,95	7,8	4,71	4,0
0,00079898	0,00386414	10,14	8,12	7,94	7,79	4,68	4,8

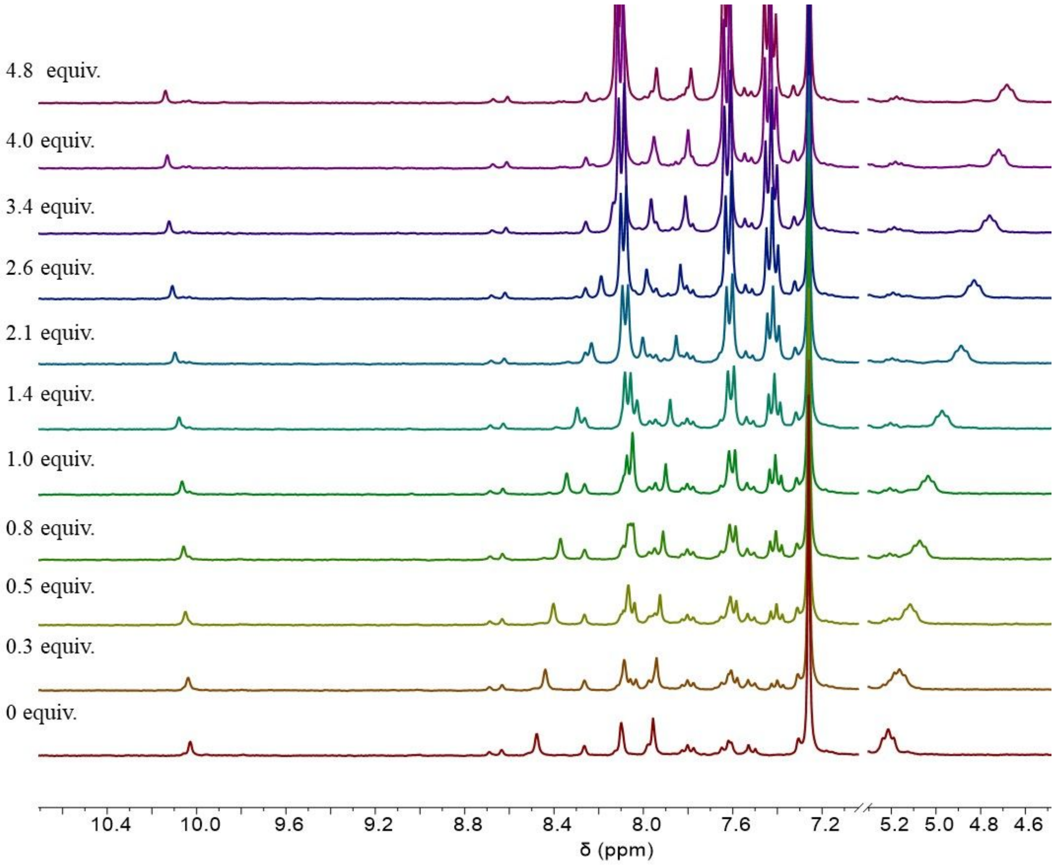

Figure S11. Selected region and spectra of the titration of complex **1** with perylene.

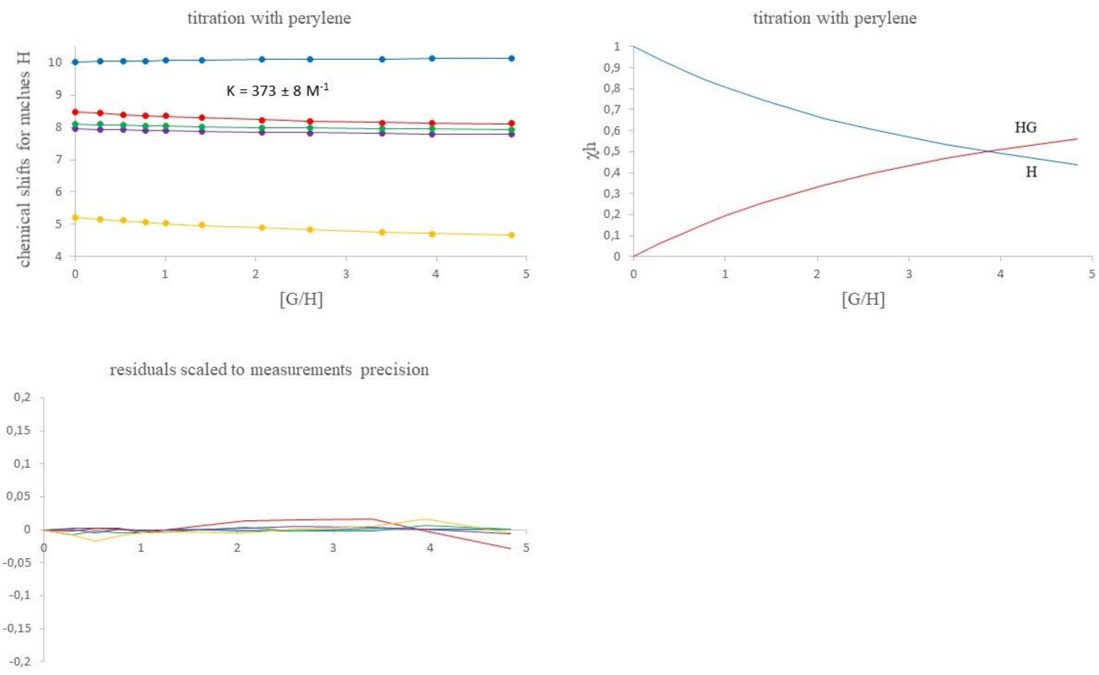

Figure S12. Non-linear least-squares fitting of the chemical shift changes of H during titration experiments of **1** with perylene. The Figure on the left represents the speciation profiles.

Titration of 1 with 3-perylenyl-methanol

Table S5. Data values from the titration study of **1** with 3-perylenyl-methanol

**[1]** M	[3-perylenyl-methanol] M	δc_H	δc_H	δc_H	δc_H	δ_{NCH2}	equiv. 3-perylenyl-methanol
0,00079898	0	10,03	8,48	8,1	7,96	5,21	0
0,00079898	0,00062668	10,09	8,3	8,04	7,89	4,95	0,8
0,00079898	0,00120693	10,13	8,18	8	7,85	4,77	1,5
0,00079898	0,00174574	10,16	8,11	7,97	7,82	4,66	2,2
0,00079898	0,00224739	10,18	8,06	7,95	7,8	4,58	2,8
0,00079898	0,00315359	10,2	8,05	7,93	7,78	4,48	3,9
0,00079898	0,00465531	10,22	7,99	7,91	7,76	4,39	5,8
0,00079898	0,00584897	10,22	7,96	7,9	7,75	4,35	7,3
0,00079898	0,00762678	10,23	7,96	7,89	7,74	4,33	9,5
0,00079898	0,0088874	10,23	7,96	7,88	7,74	4,31	11,1

0,00079898	0,01086238	10,23	7,95	7,88	7,73	4,29	13,6

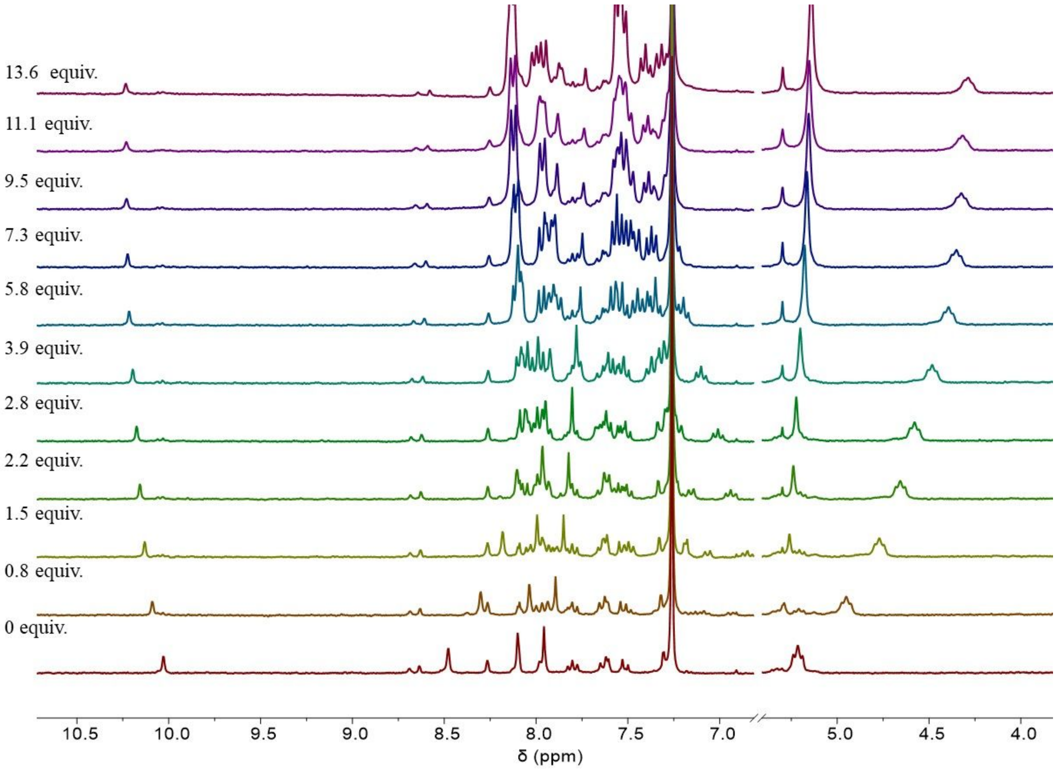

Figure S13. Selected region and spectra of the titration of complex **1** with 3-perylenyl-methanol

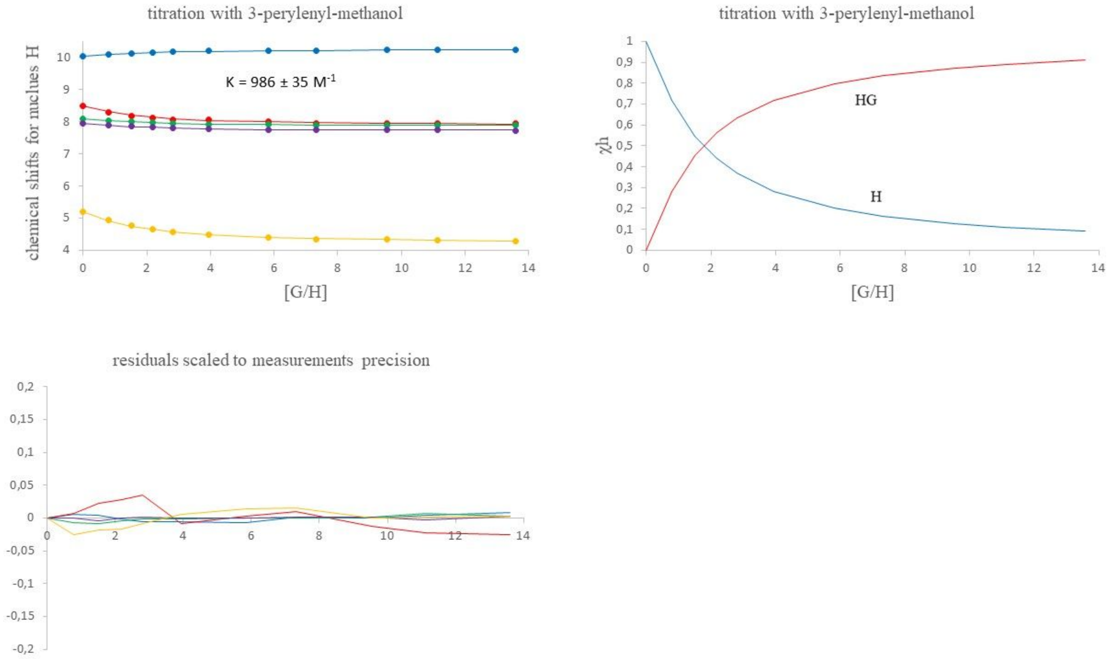

Figure S14. Non-linear least-squares fitting of the chemical shift changes of H during titration experiments of **1** with 3-perylenyl-methanol. The Figure on the left represents the speciation profiles.

Titration of 1 with coronene

Table S6. Data values from the titration study of **1** with coronene

**[1]** M	[coronene] M	δc_H	δc_H	δc_H	δc_H	δ_{NCH2}	equiv. coronene
0,00070386	0	10,03	8,48	8,1	7,96	5,22	0
0,00070386	0,00017927	10,07	8,33	8,06	7,92	5,01	0,2
0,00070386	0,00034526	10,1	8,2	8,03	7,89	4,84	0,5
0,00070386	0,0004994	10,13	8,09	7,99	7,87	4,7	0,7
0,00070386	0,00064291	10,15	8	7,97	7,85	4,58	0,9
0,00070386	0,00090214	10,18	7,86	7,94	7,82	4,4	1,3
0,00070386	0,00133174	10,22	7,7	7,89	7,78	4,18	1,9
0,00070386	0,00167321	10,25	7,6	7,86	7,76	4,05	2,4
0,00070386	0,00218178	10,28	7,49	7,83	7,73	3,91	3,1
0,00070386	0,0025424	10,29	7,44	7,82	7,72	3,83	3,6
0,00070386	0,00310738	10,3	7,4	7,81	7,71	3,79	4,4

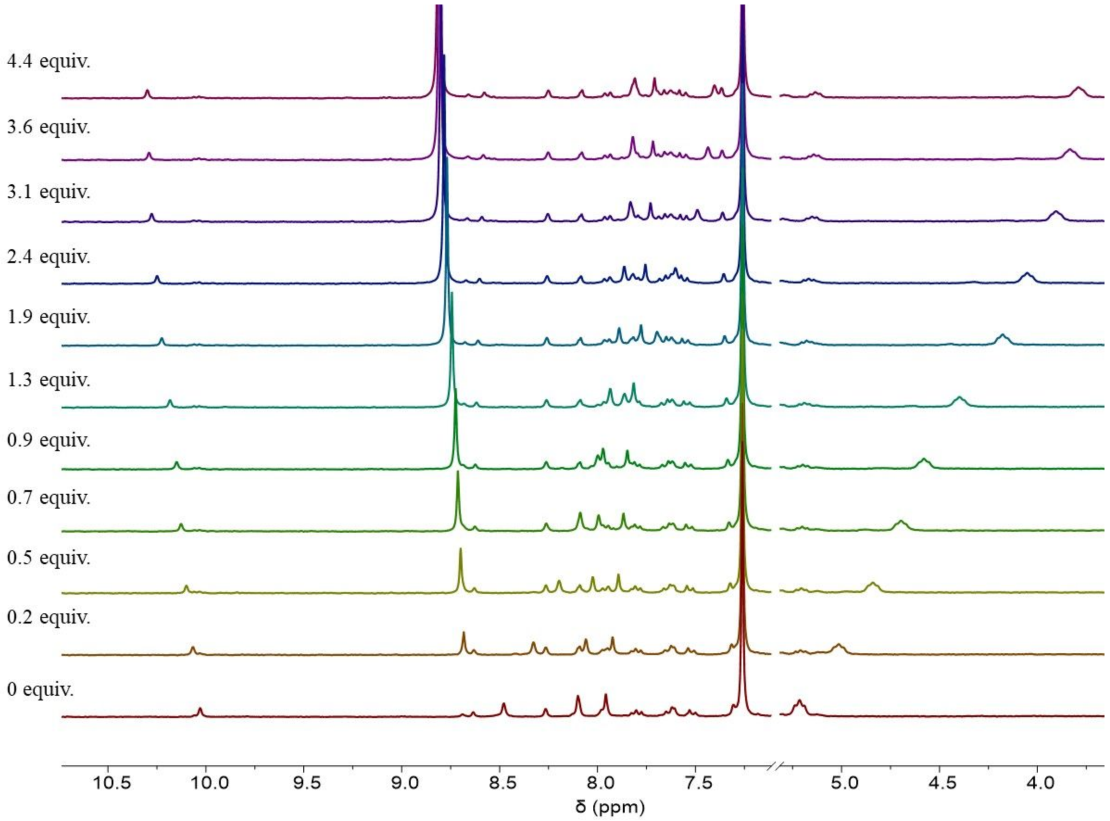

Figure S15. Selected region and spectra of the titration of complex **1** with coronene.

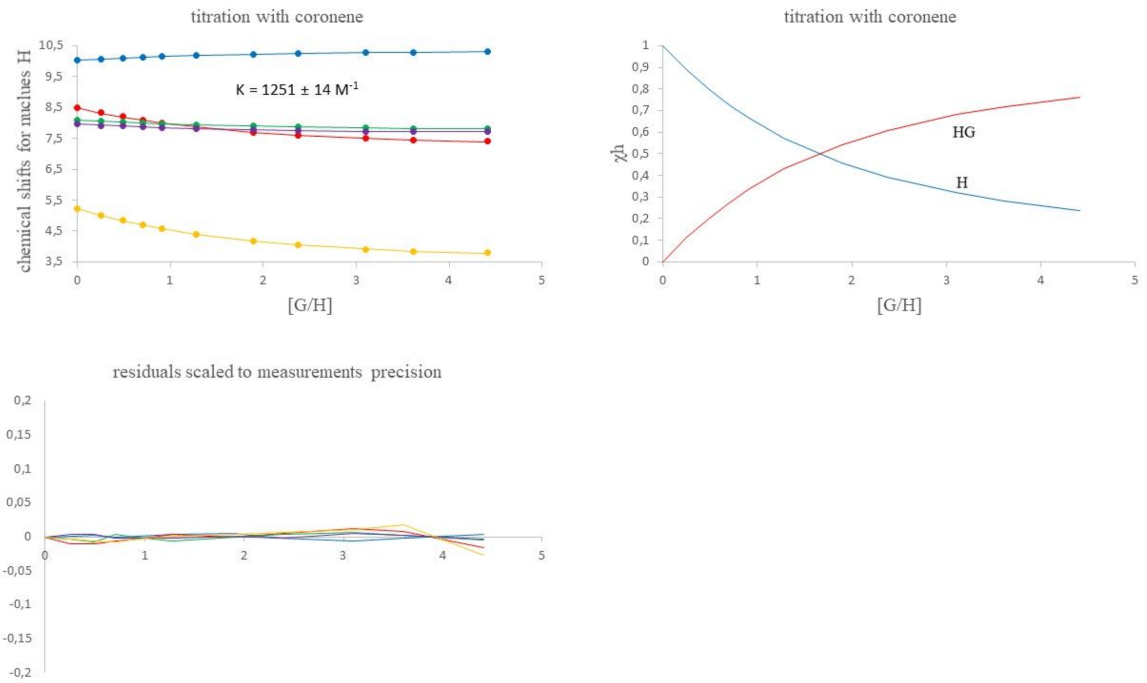

Figure S16. Non-linear least-squares fitting of the chemical shift changes of H during titration experiments of **1** with coronene. The Figure on the left represents the speciation profiles.

Titration of 1 with Au(C^N^C)(C≡CC$_6$H$_4$-OCH$_3$-p)

Table S7. Data values from the titration study of complex **1** with **12**.

[**1**] M	[Au] M	δ_{CH}	δ_{CH}	δ_{CH}	δ_{NCH2}	equiv. **12**
0,00072289	0	10,03	8,48	8,1	5,22	0
0,00072289	0,00034475	10,06	8,41	8,01	5,13	0,5
0,00072289	0,00067624	10,08	8,36	7,94	5,07	0,9
0,00072289	0,00099522	10,1	8,33	7,88	5,01	1,4
0,00072289	0,00130238	10,11	8,29	7,83	4,98	1,8
0,00072289	0,0018838	10,13	8,26	7,78	4,93	2,6
0,00072289	0,00242512	10,15	8,22	7,73	4,87	3,3
0,00072289	0,00340299	10,16	8,19	7,7	4,84	4,7
0,00072289	0,00502347	10,17	8,17	7,66	4,81	6,9
0,00072289	0,00686132	10,18	8,16	7,64	4,79	9,5
0,00072289	0,00879107	10,18	8,14	7,62	4,78	12,2

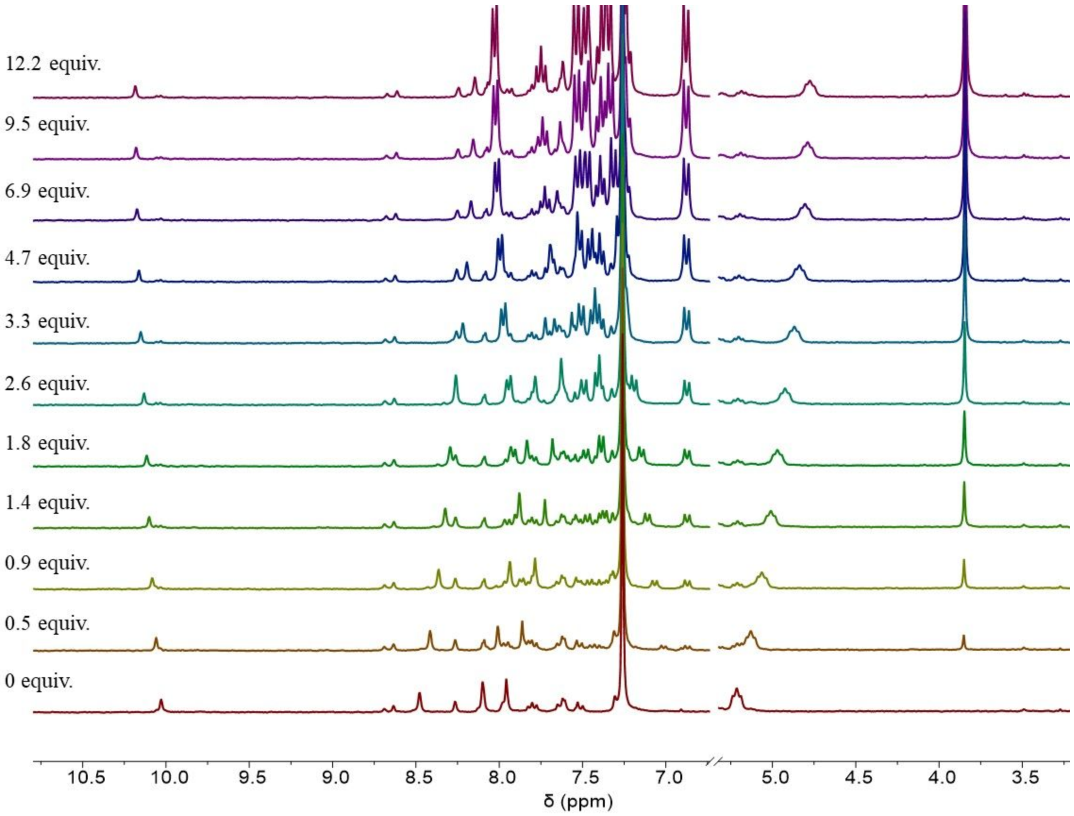

Figure S17. Selected region and spectra of the titration of complex **1** with **12**.

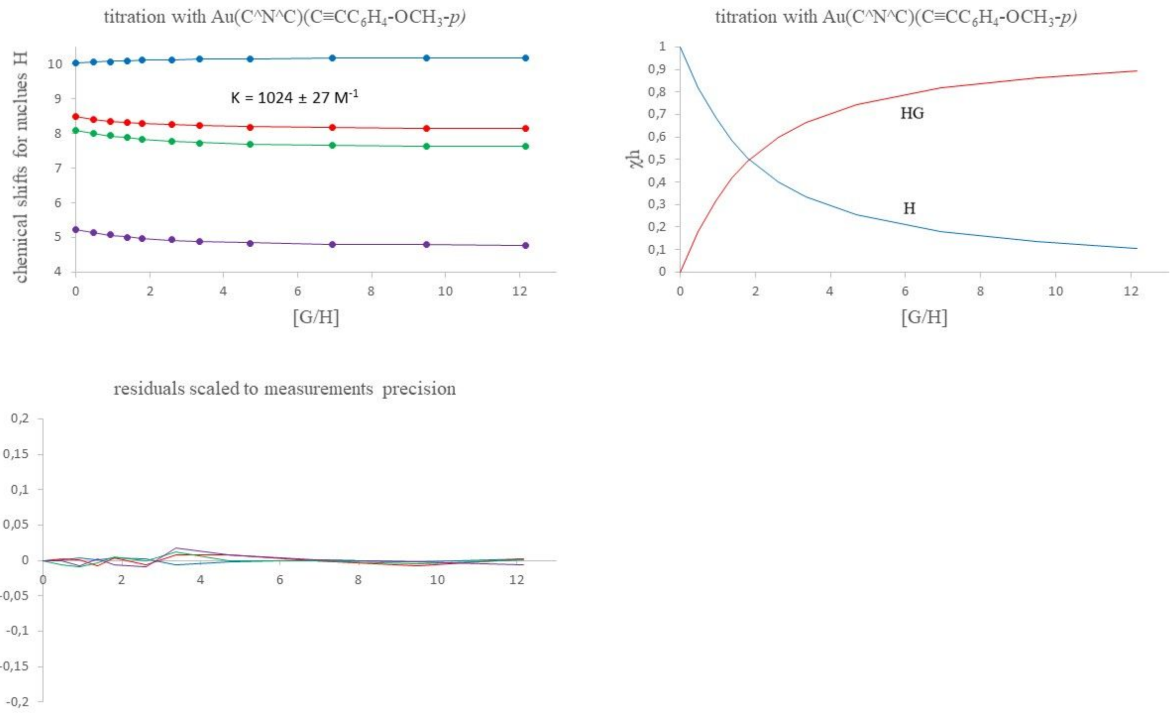

Figure S18. Non-linear least-squares fitting of the chemical shift changes of H during titration experiments of **1** with **12**. The Figure on the left represents the speciation profiles.

Titration of 1 with TNFLU

Table S8. Data values from the titration study of **1** with TNFLU

**[1]** M	[TNFLU] M	δc_H	δc_H	δc_H	δ_{NCH2}	equiv. TNFLU
0,00077995	0	8,48	8,1	7,96	5,21	0
0,00077995	0,00046035	8,39	8,05	7,85	5,12	0,6
0,00077995	0,000903	8,32	8,01	7,82	5,04	1,2
0,00077995	0,00132894	8,27	7,98	7,78	4,98	1,7
0,00077995	0,0017391	8,24	7,96	7,75	4,95	2,2
0,00077995	0,00251549	8,2	7,94	7,72	4,9	3,2
0,00077995	0,00391298	8,15	7,93	7,7	4,84	5,0
0,00077995	0,00513579	8,13	7,92	7,69	4,82	6,6
0,00077995	0,0071738	8,12	7,92	7,69	4,8	9,2
0,00077995	0,00880421	8,11	7,92	7,69	4,79	11,3
0,00077995	0,01173895	8,11	7,92	7,69	4,79	15,0

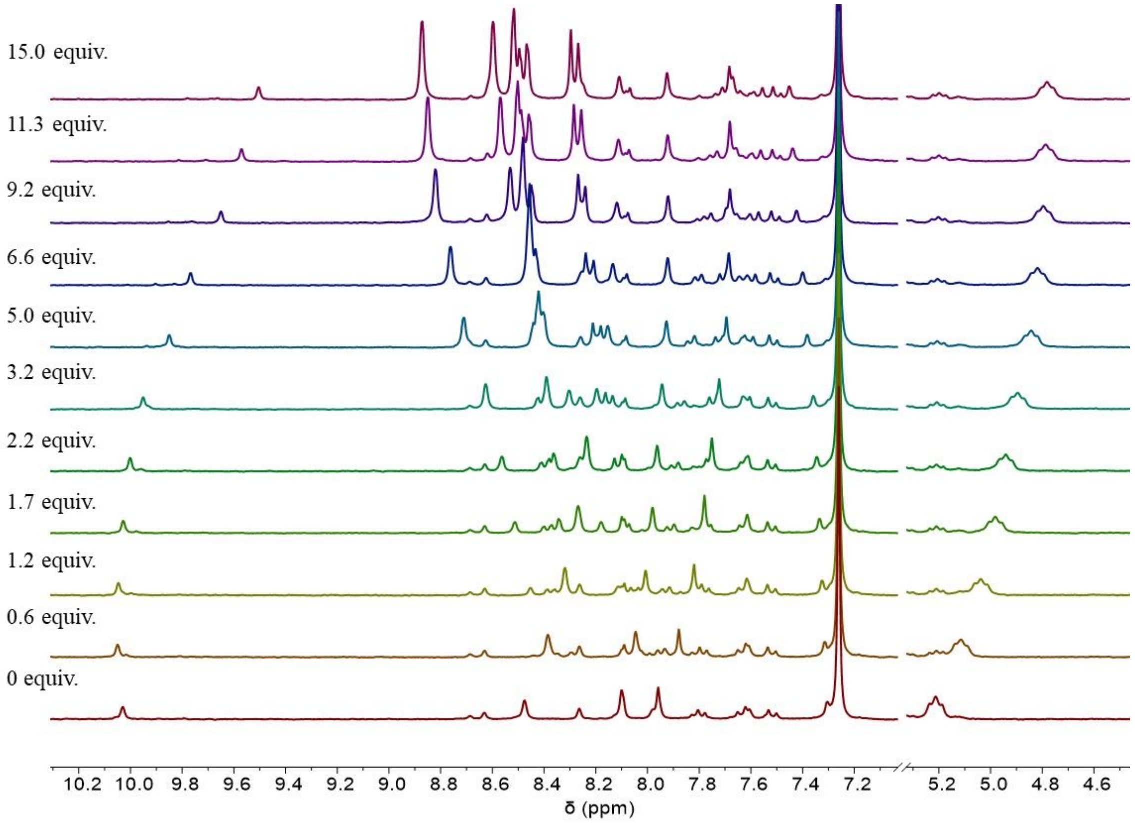

Figure S19. Selected region and spectra of the titration of complex **1** with TNFLU.

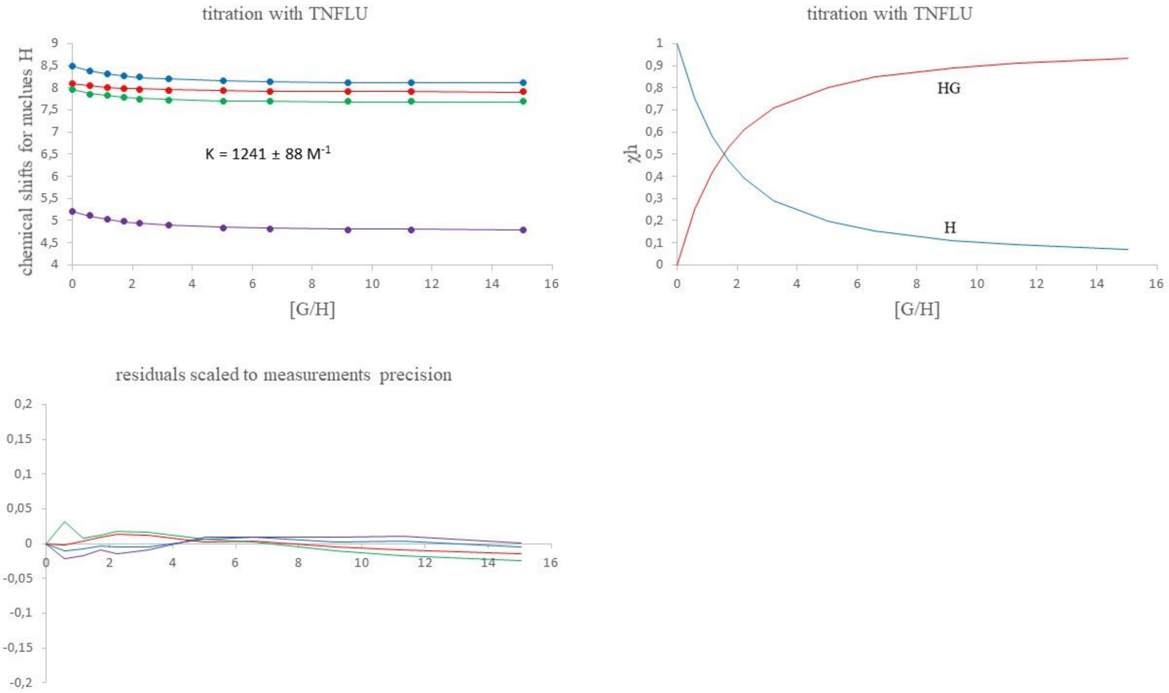

Figure S20. Non-linear least-squares fitting of the chemical shift changes of H during titration experiments of **1** with TNFLU. The Figure on the left represents the speciation profiles.

Titration of 1 with DNMFLU in $CDCl_3$.

Table S9. Data values from the titration study of complex **1** with DNMFLU.

**[1]** M	[DNMFLU] M	δ_{CH}	δ_{CH}	δ_{CH}	δ_{NCH2}	equiv. DNMFLU
0,00079898	0	8,47	8,1	7,96	5,21	0
0,00079898	0,00053555	8,43	8,08	7,93	5,17	0,7
0,00079898	0,0010505	8,4	8,06	7,9	5,13	1,3
0,00079898	0,00154602	8,36	8,04	7,88	5,1	1,9
0,00079898	0,00202318	8,34	8,03	7,86	5,07	2,5
0,00079898	0,00292639	8,31	8,01	7,84	5,03	3,7
0,00079898	0,00455216	8,26	7,99	7,8	4,98	5,7
0,00079898	0,00597471	8,23	7,97	7,77	4,94	7,5
0,00079898	0,00834562	8,19	7,94	7,74	4,89	10,4
0,00079898	0,01024235	8,18	7,93	7,72	4,87	12,8
0,00079898	0,01365647	8,16	7,92	7,7	4,86	17,1

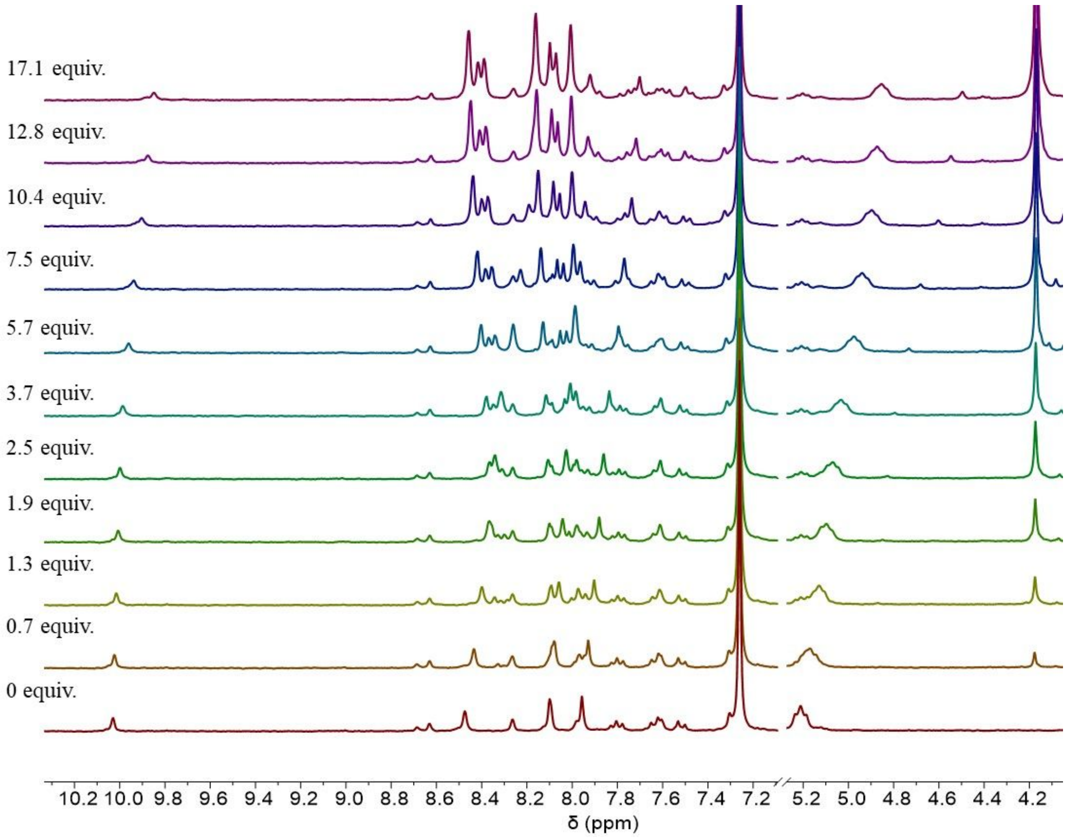

Figure S21. Selected region and spectra of the titration of complex **1** with DNMFLU.

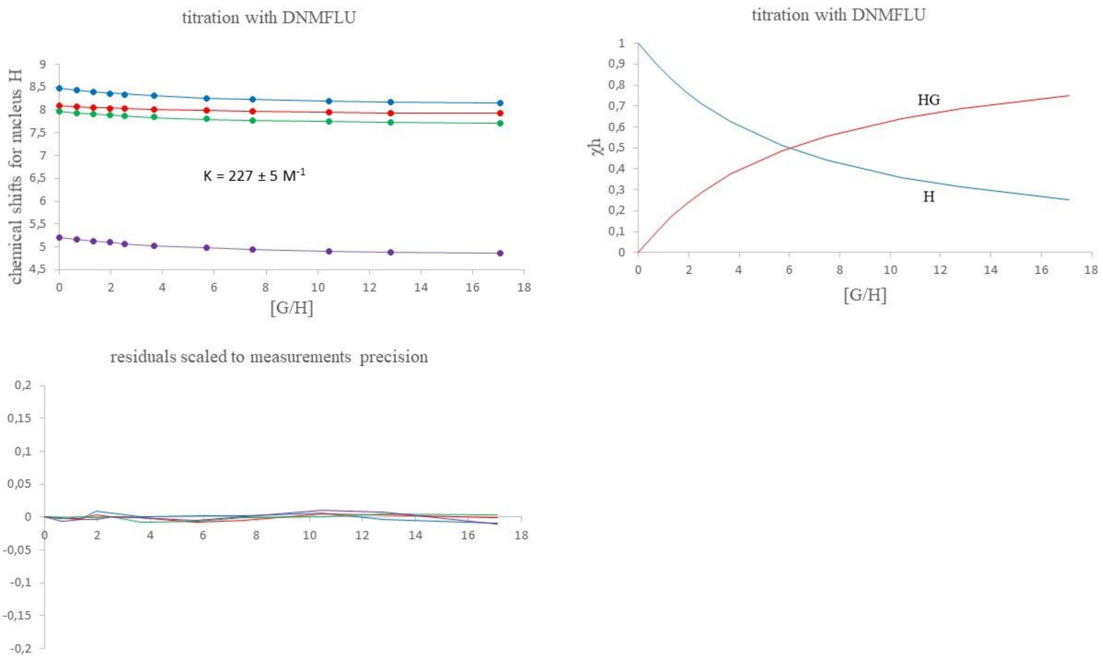

Figure S22. Non-linear least-squares fitting of the chemical shift changes of H during titration experiments of **1** with DNFLU. The Figure on the left represents the speciation profiles.

Titration of 1 with NTCDI

Table S10. Data values from the titration study of **1** with NTCDI

**[1]** M	[NTCDI] M	δc_H	δc_H	δc_H	δc_H	δ_{NCH2}	equiv. NTCDI
0,00074191	0	10,03	8,48	8,1	7,96	5,22	0
0,00074191	0,00049668	10,1	8,21	7,88	7,64	5,06	0,7
0,00074191	0,00095657	10,12	8,11	7,8	7,53	5	1,3
0,00074191	0,00138362	10,12	8,08	7,77	7,48	4,98	1,9
0,00074191	0,00178121	10,12	8,05	7,76	7,45	4,96	2,4
0,00074191	0,00249944	10,13	8,04	7,75	7,43	4,95	3,4
0,00074191	0,00368964	10,13	8,03	7,73	7,42	4,95	5,0
0,00074191	0,00463571	10,13	8,02	7,72	7,41	4,95	6,2
0,00074191	0,00604473	10,13	8,01	7,71	7,41	4,95	8,1
0,00074191	0,00704386	10,13	8,01	7,71	7,4	4,95	9,5
0,00074191	0,00860917	10,13	8,01	7,71	7,4	4,95	11,6

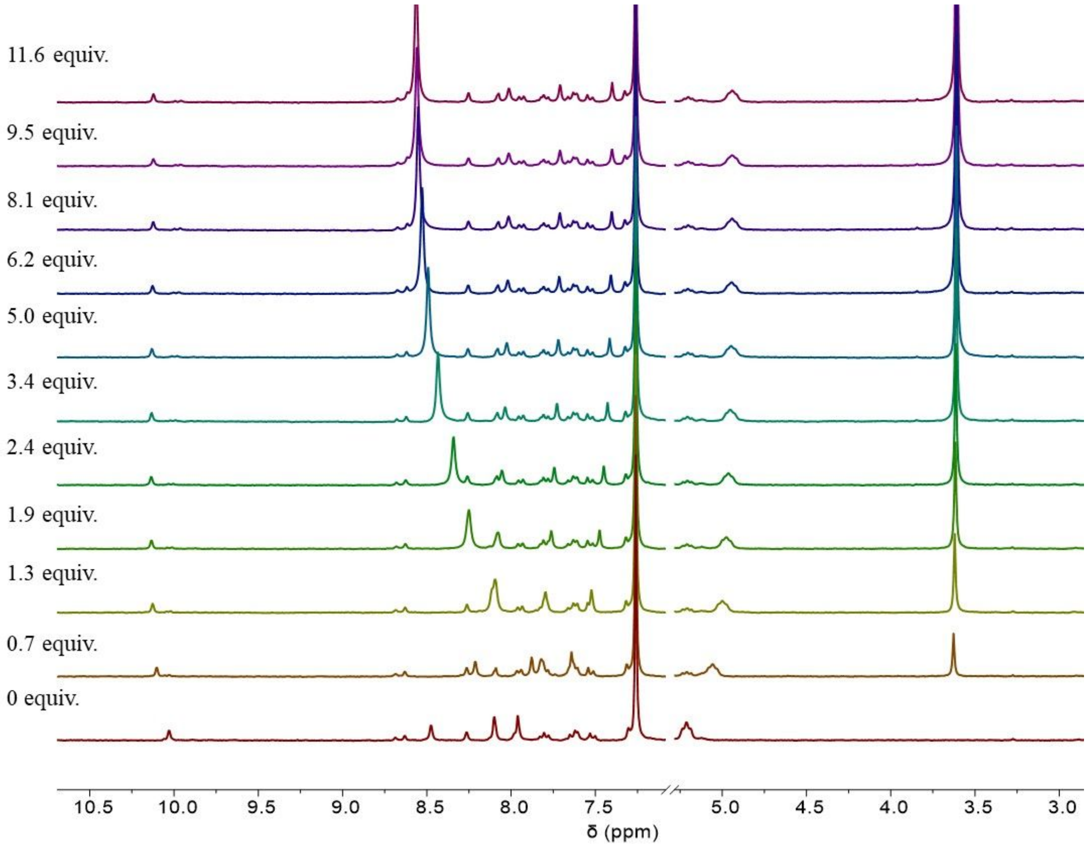

Figure S23. Selected region and spectra of the titration of complex **1** with NTCDI.

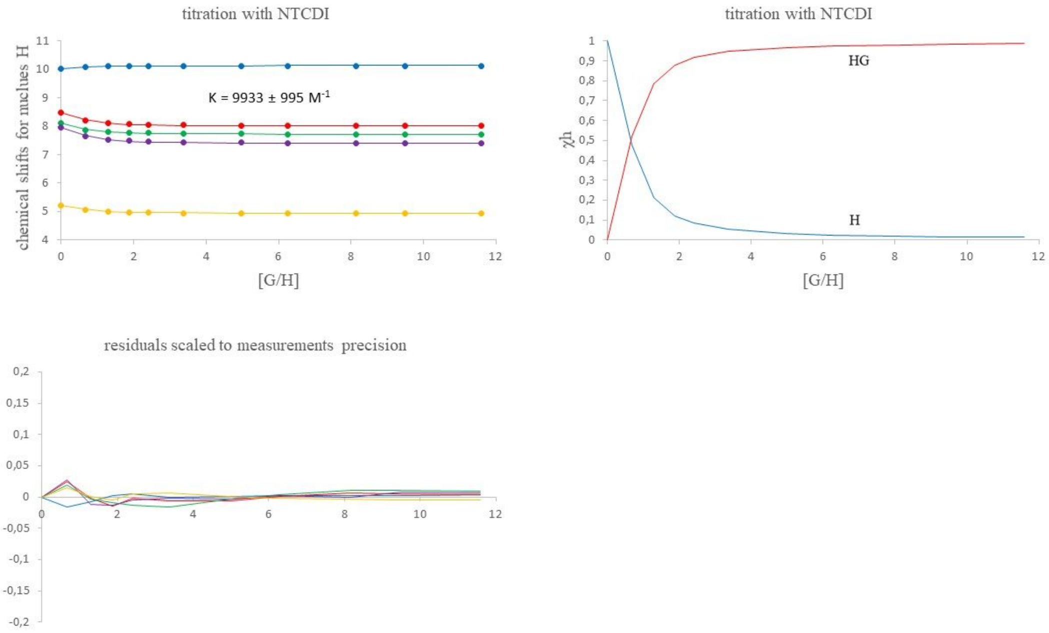

Figure S24. Non-linear least-squares fitting of the chemical shift changes of H during titration experiments of **1** with NTCDI. The Figure on the left represents the speciation profiles.

Titration of 2 with perylene in $CDCl_3$.

Table S11. Data values from the titration study of complex **2** with perylene.

[**2**] M	[perylene] M	δ_{NH}	δ_{CH}	δ_{CH}	δ_{CH}	δ_{NCH2}	equiv. perylene
0,00048403	0	9,08	8,59	8,19	8,04	5,27	0
0,00048403	0,0002134	9,08	8,58	8,18	8,03	5,26	0,4
0,00048403	0,000411	9,09	8,57	8,18	8,03	5,24	0,8
0,00048403	0,00059448	9,09	8,56	8,18	8,02	5,23	1,2
0,00048403	0,00076531	9,09	8,55	8,16	8,02	5,22	1,6
0,00048403	0,00092475	9,1	8,54	8,16	8,01	5,21	1,9
0,00048403	0,00107391	9,1	8,53	8,15	8,01	5,19	2,2
0,00048403	0,00134509	9,1	8,52	8,15	8	5,18	2,8
0,00048403	0,00158529	9,1	8,51	8,15	8	5,16	3,3
0,00048403	0,00199177	9,11	8,48	8,14	7,99	5,13	4,1
0,00048403	0,00232263	9,11	8,47	8,14	7,98	5,11	4,8
0,00048403	0,00282865	9,12	8,45	8,13	7,97	5,09	5,8

0,00048403	0,00319745	9,12	8,43	8,13	7,96	5,06	6,6
0,00048403	0,00369901	9,13	8,41	8,12	7,96	5,04	7,6

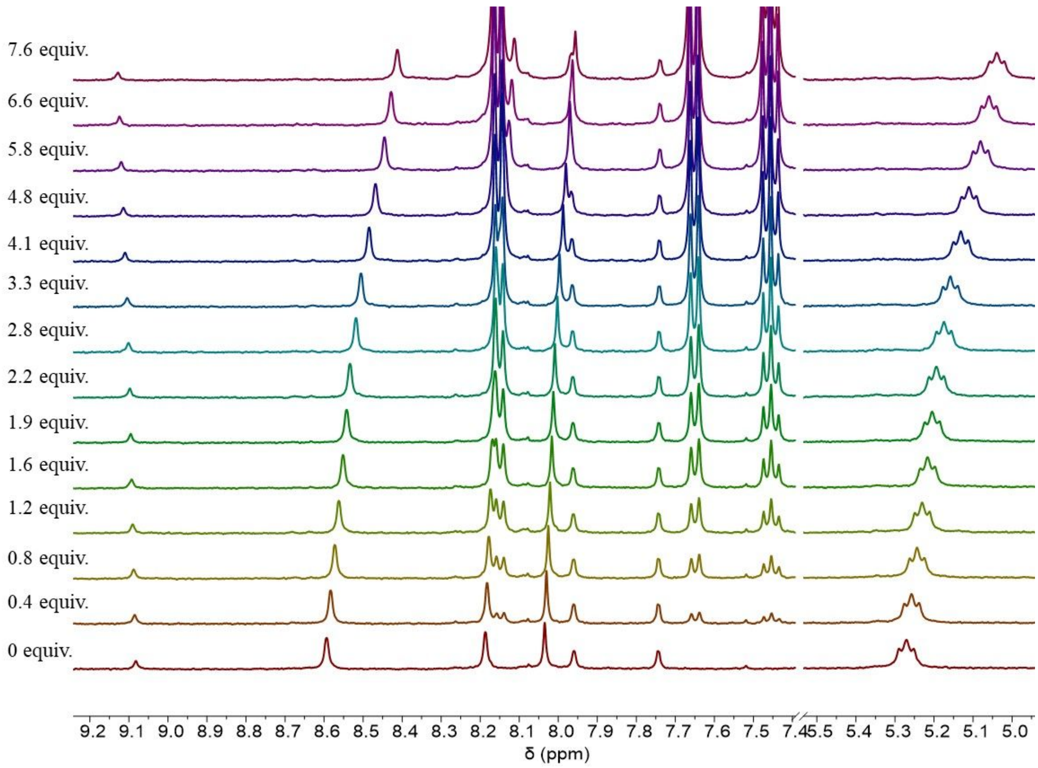

Figure S25. Selected region and spectra of the titration of complex **2** with perylene.

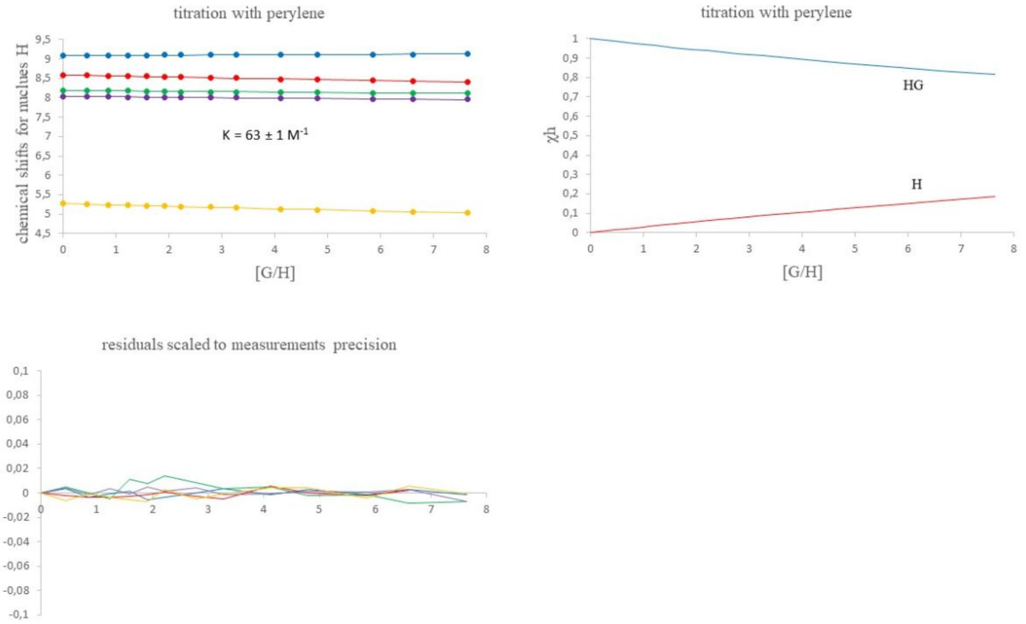

Figure S26. Non-linear least-squares fitting of the chemical shift changes of H during titration experiments of **2** with perylene. The Figure on the left represents the speciation profiles.

*Titration of **2** with coronene in $CDCl_3$*

Table S12. Data values from the titration study of complex **2** with coronene.

[**2**] M	[coronene] M	δ_{NH}	δ_{CH}	δ_{CH}	δ_{CH}	δ_{NCH2}	equiv. coronene
0,00052437	0	9,08	8,59	8,19	8,04	5,27	0
0,00052437	0,00010761	9,09	8,57	8,18	8,03	5,25	0,2
0,00052437	0,00020724	9,1	8,55	8,17	8,02	5,22	0,4
0,00052437	0,00029976	9,1	8,53	8,16	8,02	5,19	0,6
0,00052437	0,0003859	9,11	8,51	8,16	8,01	5,17	0,7
0,00052437	0,00046629	9,11	8,49	8,15	8,01	5,15	0,9
0,00052437	0,0005415	9,12	8,47	8,15	8	5,13	1,0
0,00052437	0,00067825	9,13	8,44	8,14	7,99	5,09	1,3
0,00052437	0,00079936	9,14	8,42	8,13	7,98	5,06	1,5
0,00052437	0,00100433	9,15	8,37	8,11	7,97	5,01	1,9
0,00052437	0,00117116	9,16	8,34	8,1	7,96	4,97	2,2
0,00052437	0,00142631	9,17	8,3	8,09	7,94	4,92	2,7
0,00052437	0,00161227	9,18	8,26	8,08	7,94	4,88	3,1
0,00052437	0,00186518	9,19	8,23	8,07	7,93	4,85	3,6

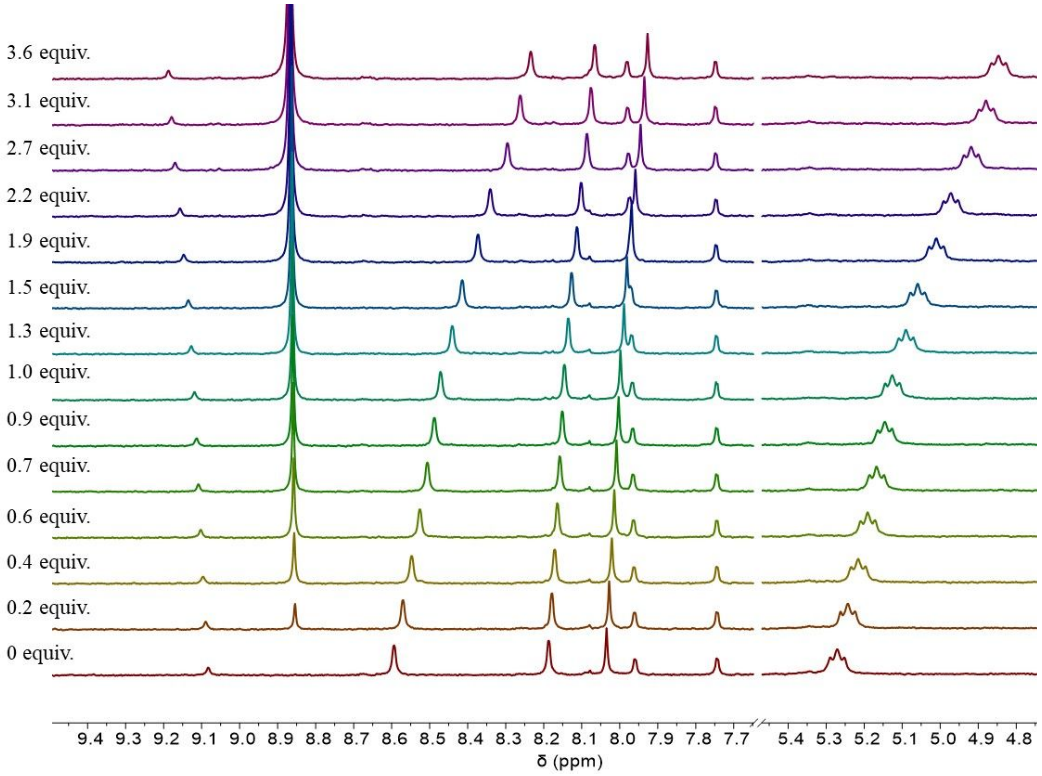

Figure S27. Selected region and spectra of the titration of complex **2** with coronene.

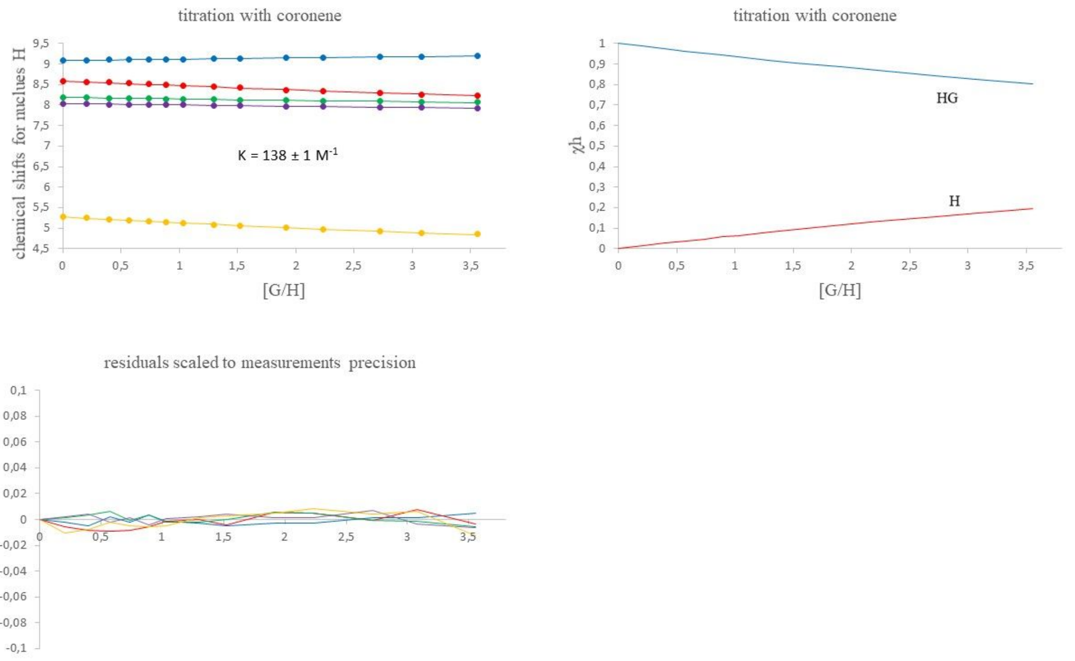

Figure S28. Non-linear least-squares fitting of the chemical shift changes of H during titration experiments of **2** with coronene. The Figure on the left represents the speciation profiles.

Titration of 2 with TNFLU in $CDCl_3$

Table S13. Data values from the titration study of complex **2** with TNFLU.

**[2]** M	[TNFLU] M	δ_{NH}	δ_{CH}	δ_{CH}	δ_{CH}	δ_{NCH2}	equiv. TNFLU
0,00052437	0	9,08	8,6	8,19	8,04	5,27	0
0,00052437	0,0002513	9,21	8,54	8,16	7,99	5,22	0,5
0,00052437	0,00049768	9,32	8,5	8,13	7,95	5,17	0,9
0,00052437	0,00073927	9,42	8,46	8,1	7,92	5,13	1,4
0,00052437	0,00097621	9,5	8,43	8,09	7,89	5,09	1,9
0,00052437	0,00143669	9,59	8,39	8,06	7,85	5,05	2,7
0,00052437	0,00188011	9,66	8,36	8,04	7,82	5,02	3,6
0,00052437	0,00271945	9,75	8,31	8,01	7,78	4,97	5,2
0,00052437	0,0035009	9,82	8,28	7,99	7,75	4,94	6,7
0,00052437	0,00491255	9,89	8,22	7,96	7,71	4,9	9,4
0,00052437	0,00725186	9,95	8,19	7,93	7,66	4,85	13,8
0,00052437	0,00911131	9,96	8,17	7,92	7,64	4,83	17,4
0,00052437	0,0109602	9,96	8,15	7,91	7,63	4,82	20,9
0,00052437	0,01269076	9,96	8,15	7,91	7,63	4,82	24,2

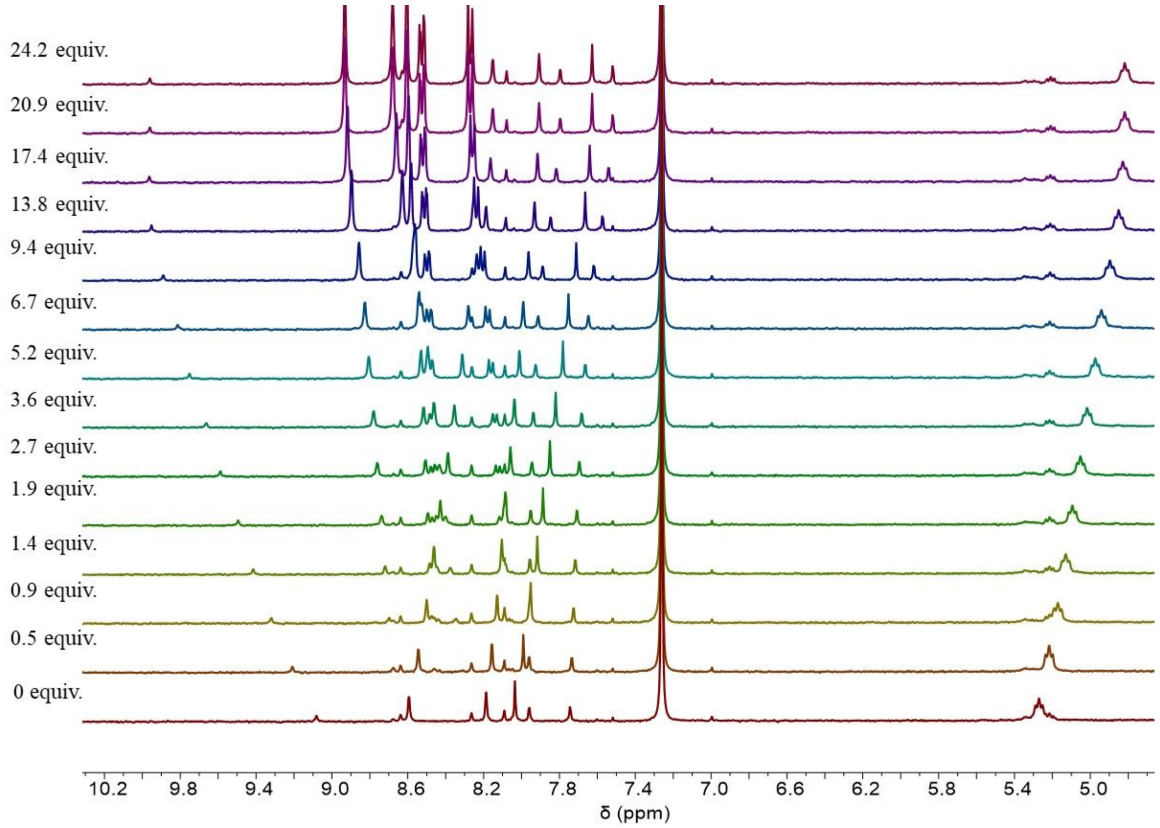

Figure S29. Selected region and spectra of the titration of complex **2** with TNFLU.

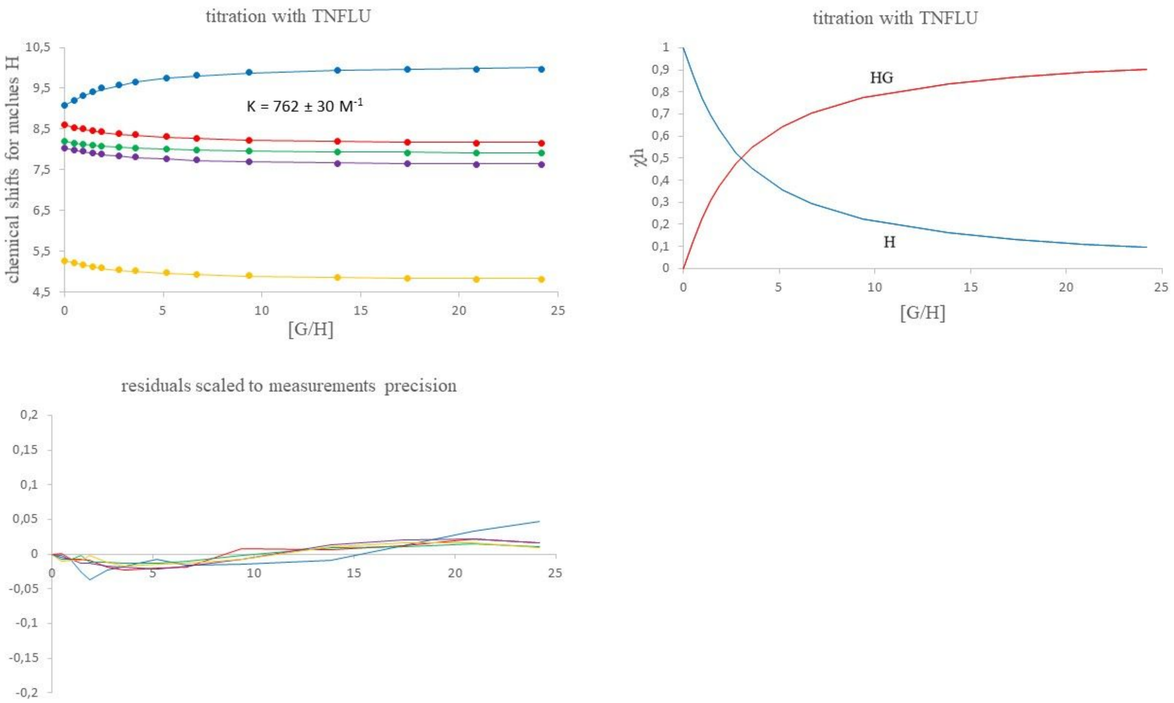

Figure S30. Non-linear least-squares fitting of the chemical shift changes of H during titration experiments of **2** with TNFLU. The Figure on the left represents the speciation profiles.

4. DOSY experiments

The experiments were carried out in $CDCl_3$, at constant concentrations of 5 mM.

Complexes	G (m^2/s)
**1**[7]	5.75 10^{-10}
**NTCDI@1**	5.34 10^{-10}
**12@1**	5.00 10^{-10}

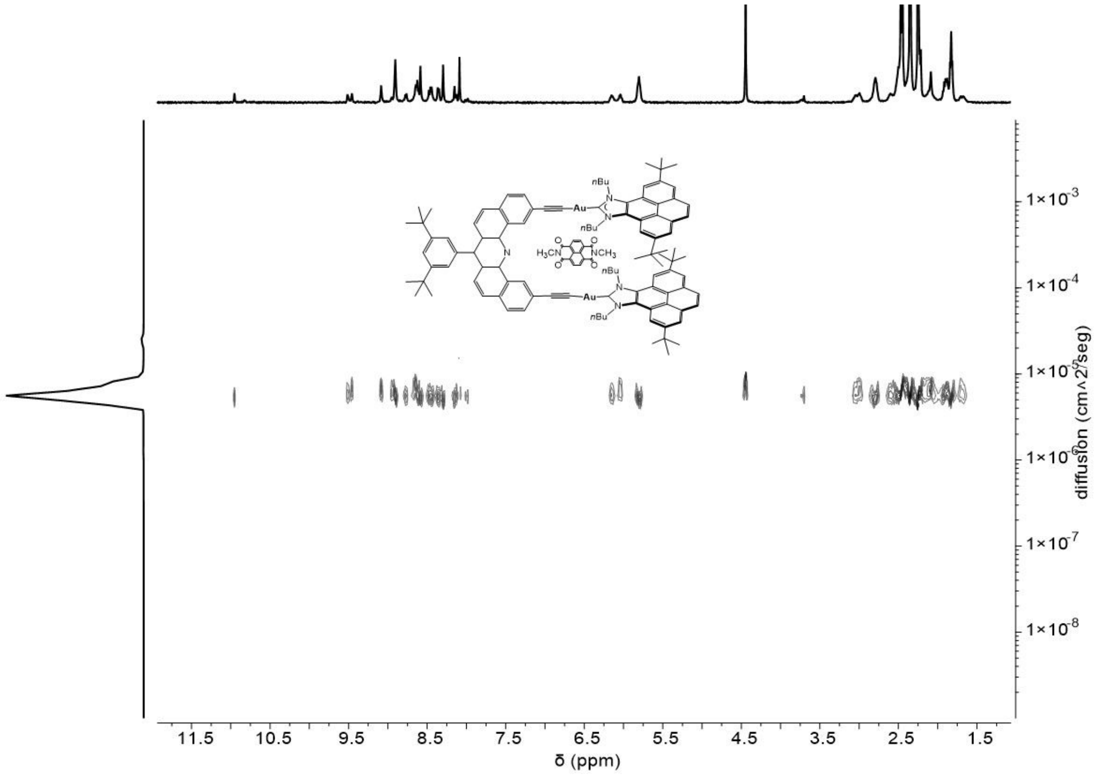

Figure S31. DOSY NMR spectrum of **NTCDI@1**.

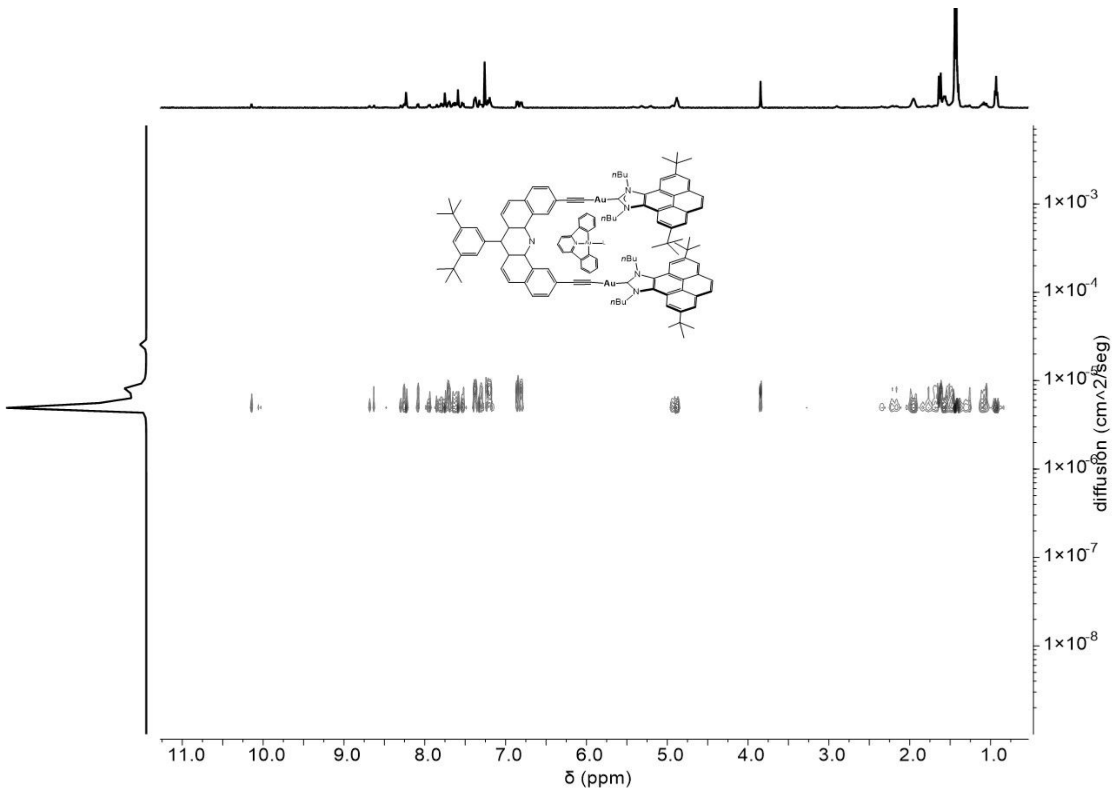

Figure S32. DOSY NMR spectrum of **12@1**.

5. References

(1) Ibáñez, S.; Poyatos, M.; Peris, E. Gold Catalysts with Polyaromatic-NHC ligands. Enhancement of Activity by Addition of Pyrene. *Organometallics* **2017**, *36*, 1447-1451.

(2) Kim, D.; Jung, I.; Ho Song, K.; Ooh Kang, S.; Ko, J. *Journal of Organometallics Chemistry* **2006**, *691*, 5946-5954.

(3) Liang, Y.; Zhang, P.; J., C. *Chem. Sci.* **2013**, *4*, 1330-1337.

(4) Ibáñez, S.; Peris, E. *Chem. Eur. J.* **2021**, *27*, 9661-9665.

(5) Wong, K. M. C.; Hung, L. L.; Lam, W. H.; Zhu, N.; Yam, V. W. W. *J. Am. Chem. Soc.* **2007**, *129*, 4350-4365.

(6) Biz, C.; Ibañez, S.; Poyatos, M.; Gusev, D.; Peris, E. Gold(I) Metallo-Tweezers for the Recognition of Functionalized Polycyclic Aromatic Hydrocarbons by Combined pi-pi Stacking and H-Bonding. *Chem. Eur. J.* **2017**, *23*, 14439-14444.

(7) Ibáñez, S.; Vicent, C.; Peris, E. Clippane: A Mechanically Interlocked Molecule (MIM) Based on Molecular Tweezers. *Angew. Chem., Int. Ed.* **2022**, *61*, e202112513
